# Label Retention Identifies a Multipotent Mesenchymal Stem Cell-Like Population in the Postnatal Thymus

**DOI:** 10.1371/journal.pone.0083024

**Published:** 2013-12-10

**Authors:** Masako Osada, Varan J. Singh, Kenmin Wu, Derek B. Sant’Angelo, Mark Pezzano

**Affiliations:** 1 Department of Biology, The City College of New York, CUNY, New York, New York, United States of America; 2 Child Health Institute of New Jersey, Department of Pediatrics, Rutgers, Robert Wood Johnson Medical School, New Brunswick, New Jersey, United States of America; University Hospital Heidelberg, Germany

## Abstract

Thymic microenvironments are essential for the proper development and selection of T cells critical for a functional and self-tolerant adaptive immune response. While significant turnover occurs, it is unclear whether populations of adult stem cells contribute to the maintenance of postnatal thymic epithelial microenvironments. Here, the slow cycling characteristic of stem cells and their property of label-retention were used to identify a K5-expressing thymic stromal cell population capable of generating clonal cell lines that retain the capacity to differentiate into a number of mesenchymal lineages including adipocytes, chondrocytes and osteoblasts suggesting a mesenchymal stem cell-like phenotype. Using cell surface analysis both culture expanded LRCs and clonal thymic mesenchymal cell lines were found to express Sca1, PDGFRα, PDGFRβ,CD29, CD44, CD49F, and CD90 similar to MSCs. Sorted GFP-expressing stroma, that give rise to TMSC lines, contribute to thymic architecture when reaggregated with fetal stroma and transplanted under the kidney capsule of nude mice. Together these results show that the postnatal thymus contains a population of mesenchymal stem cells that can be maintained in culture and suggests they may contribute to the maintenance of functional thymic microenvironments.

## Introduction

The thymus is responsible for the generation of new T cells from hematopoietic stem cells (HSC) and the selection of T cells expressing a functional self-tolerant T cell receptor (TCR). Unique thymic epithelial microenvironments in the thymic stroma control these critical processes [1]. The thymic stroma is broadly divided into two distinct regions called the cortex and the medulla. Cortical TECs (cTECs) are responsible for the attraction of T cell precursors, commitment to the T cell lineage, expansion of immature double-negative (DN) thymocytes and positive selection of double positive (DP) thymocytes [2]. Medullary thymic epithelial cells (mTECs) are a heterogeneous population of cells that create a microenvironment necessary for the maturation of CD4 and CD8 single positive (SP) thymocytes. mTECs express a wide array of tissue restricted antigens (TRAs) [3-5] that, when presented in the context of major histocompatibility complex class II (MHCII)^+^ on mTECs and dendritic cells, function to negatively select thymocytes that bear high affinity self-reactive TCRs [6]. In addition to TECs, mesenchymal cells have also been shown to be important for the initial development and subsequent maintenance of a functional thymic microenvironment [7,8]. 

In adulthood, thymic atrophy results in a progressive loss of normal thymic cortical and medullary epithelial architecture and a subsequent reduced capacity to generate mature T cells. This atrophy involves a transition to a thymus enriched in fibroblasts and ultimately adipocytes, which appear to arise through epithelial mesenchyme transition [9-11]. Interestingly, thymic atrophy appears to be reversible, allowing normal thymic architecture and T cell output to be restored [12]. These studies show a plasticity of the thymic architecture and suggest that TEC progenitors persist in the adult thymus and can be activated to proliferate and differentiate later in life. 

Stem cells (SCs) are unique in their ability to self-renew and to differentiate into the cell lineages that make up their tissue of origin. Stem cells with the capacity to regenerate their specific tissue of origin have been identified in numerous adult tissues, including the bone marrow, epidermis, hair follicle, intestine, brain, testis and cornea [13-15]. Another class of multipotent or potentially pluripotent cells, the mesenchymal stem cells (MSCs) are resident in virtually all postnatal tissues and organs [16-19]. 

Due to their minority status in adult tissues and a limited number of defining cell surface markers, identification of adult stem cells has relied primarily on anatomical characteristics and their slow cycling properties. Recently, however, Tumbar et al [20] utilized a novel H2BGFP transgenic system to mark infrequently cycling cells of adult skin epithelium. These label-retaining cells (LRCs) were shown to be stem cells. In this study we used this transgenic model to identify a population of LRCs in the postnatal thymus. These LRCs, and clonal lines derived from sorted thymic stroma with the same surface characteristics, exhibited enhanced *in vitro* growth potential and, when subjected to different culture conditions, were found to have retained the capacity to differentiate into adipocytes, chondrocytes and osteocytes. This multipotentiality is highly reminiscent of mesenchymal stem cells. When sorted populations of these cells were reaggregated with fetal stroma and transferred under the kidney capsule they contributed to the resulting thymus as TECs. Therefore, our studies demonstrate previously unrecognized developmental plasticity for EpCAM–expressing cells in the postnatal thymus and suggest that TMSCs may contribute to maintenance of postnatal thymic microenvironments critical to T cell development. 

## Materials and Methods

### Ethics Statement

All mice used in this study were bred and maintained at the City College of New York animal facility and all experiments were performed with approval from the City College of New York Institutional Animal Care and Use Committee. The animal care facility at the City College of New York is certified at both the State and Federal levels. The CCNY animal assurance number is A3733-01. 

### Mice

Dr. Adam Glick (Penn State University) generously provided *K5t*TA mice. *TetO-H2BGFP* mice have been previously described [21-24]. TetO-H2BGFP, Nude, and *C57BL/6JJ* mice were obtained from the Jackson Laboratory (Bar Harbor, ME). Adult *TetO-H2BGFP*;*K5t*TA DT and litter mate heterozygous control animals were fed mouse diet containing doxycycline (Dox) (2 g/kg, BioServe, NJ) for 8 weeks to 6 months beginning at 4 weeks of age, unless otherwise indicated. 

### Antibodies

The following primary antibodies were used for experiments: CD45-PE Cy7, CD45-APC Cy7 (clone 30-F11, BD Bioscience), I-A/I-E-APC (clone M5/114.15.2, BD Bioscience), cytokeratin 5 (MK-5, Covance), cytokeratin 14 (MK-14, Covance), Troma I (Developmental Studies Hybridoma Bank, IA), MTS10 (kindly provided by Dr. Richard Boyd from Monash University, Australia), ΔNP63 (clone N-16, Santa Cruz), Aire (clone M-300, Santa Cruz), EpCAM-PE (clone G8.8, eBioscience), Ki67-FITC (clone B56, BD Bioscience), Ki67 (clone SP6, LabVision), Sca1-PE Cy7, Sca1-APC Cy7(clone D7, eBioscience), CD49F-Biotin (eBioGoH3, eBioscience), CD29-FITC, CD29-PE Cy7(clone Ha2/5, eBioscience) , Laminin (Millipore). CD106-FITC (BD Bioscience), CD140a-Biotin(clone APA5, eBioscience), CD140b-PE (clone APB5, eBioscience), CD44-APC (clone IM7, BD Bioscience), CD90.2(clone 30-H12, BD Bioscience), CD34-PE (clone RAM#4, eBioscience), SSEA1(clone MC480, Stem Cell Tecnologies), Rabbit anti-GFP (Life), CD80 (eBioscience), and CD205 (LY75/DEC-205) clone HD30 (Millipore), Rat IgG2a isotype-PE, Rat IgG2a isotype-FITC, Rat IgG2a isotype-PE-Cy7, Rat IgG2a isotype-PerCP Cy5.5, Rat IgG2a isotype-APC Cy7, Rat IgG2a isotype-APC and Rat IgG2a isotype-Biotin (BD Bioscience). The following secondary reagents were used for experiments: donkey anti rabbit IgG-TRITC, donkey anti rabbit IgG-Cy5, donkey anti rabbit IgG-FITC, donkey anti rat IgG-TRITC, donkey anti goat IgG-FITC, goat anti rat IgM-TRITC (Jackson ImmunoResearch), anti-rat IgG2a-FITC, anti-rat IgM-FITC, stretavidin-APC, stretavidin-APC Cy7, streptavidin-PerCP Cy5.5 (BD Bioscience) and stretavidin-TRITC (Southern Biotechnology Associate). 

### Thymic stromal cell preparation

Embryonic thymi were digested with Dispase (1 mg/mL) and Deoxyribonuclease I solution (1 μg/mL), (Stem Cell Technologies) at 37°C for 15 minutes with occasional gentle agitation with a glass Pasteur pipette. The resulting single cell suspension was washed with PBS and passed through 100μm strainer (BD Bioscience) to remove any remaining undigested tissue. Adult thymi were cut into small pieces and the majority of thymocytes were released by gentle agitation using a glass Pasteur pipette. The resulting tissue fragments were digested with Collagenase/Hyaluronidase in Dulbecco’s Modified Eagle’s Medium (Stem Cell Technologies) for 15 minutes at 37°C followed by Dispase (1 mg/mL) and Deoxyribonuclease I solution (0.1 μg/mL), (Stem Cell Technologies) for 5 minutes at 37°C. The single cell suspension was washed with PBS and passed through a 100μm strainer (BD Bioscience). 

### Flow cytometery

Cells were suspended in 100 μl of FACS staining buffer (FSB-1% fetal bovine serum, 5 mM EDTA and 0.02% NaN_3_ in PBS) with appropriately diluted primary antibodies for 20 minutes on ice in the dark. Secondary antibodies appropriately diluted in FSB were added cells were incubated for an additional 20 minutes on ice in the dark. After washing, cells were resuspended in 500 μl of FSB for data acquisition. Live/dead discrimination was applied using ToPro3 (Invitrogen). Data acquisition was performed using an LSRII analyzer complete with three lasers (BD Bioscience) and cell sorting was performed using a FACS Aria (BD Bioscience). FACS data was analyzed using Flow Jo software (Tree Star) or FACS Diva software (BD Bioscience).

### Immunohistochemistry and confocal microscopy

Fresh tissues were embedded in OCT medium (Fisher); snap frozen and sectioned (8μm) using a Leica CM1950 Cryostat. Sections were air dried on bond-rite slides and then fixed in 4% paraformaldehyde followed by 100% ice-cold acetone. Sections were washed with PBS and blocked with blocking buffer (1% BSA, 0.1%Triton-X, 5% normal serum in PBS) for 10 min. Sections were incubated with appropriately diluted primary antibodies in blocking buffer in a humidified chamber for 1 hour at 37°C followed by incubation with secondary reagents diluted in blocking buffer in humidified chamber for 30 minutes at 37°C, then mounted with ProLong gold anti-fade reagent with DAPI (Invitrogen). Isotype control staining was performed for all primary antibodies to ensure specificity of staining. Images were acquired using Zeiss LSM510 confocal microscope and analyzed using LSM software (Zeiss).

### RNA isolation and gene expression analysis

Total RNA was isolated from sorted EpCAM^+^ thymic epithelial cells or cultured cells using Trizol reagent (Invitrogen). RT-PCR was performed using SuperScript III first-strand synthesis system (Invitrogen). End point PCR gene expression analysis of sorted TECs and TMSC clonal lines was performed using a BioRad MyCycler. The cycling condition was 95°C for 2 minutes for the initial denature followed by 95°C 30 seconds, 55°C 30 seconds and 72°C 45 seconds for 40 cycles with the final extension of 5 minutes at 72°C. The amplified PCR products were visualized on 2% agrose gel containing 0.5μg/mL ethidium bromide and images were captured using a Kodak Gel Logic 100 for documentation. The primers used for PCR analysis of gene expression were as follows, listed 5’-3’:

UTF1 F-GCTCCCCAGTCGTTGAATACC, R-CCAGAGAAACGGTTTGGTCG[25] 

FoxD3 F-CCATCCCCTCACTCACCTAAGC, R-AAAGAATGTCCCTCCCACCC[25]

Lgr5 F -CTTCCGAATCGTCGATCTTC, R-AACGATCGCTCTCAGGCTAA[26]

FoxA1 F-CTGCAAATGATCAGGAACATAATCC

R-CGTGGTGTTAGTTTTAGACAAACGG[25]

Cdx1 F- GCACAAGGATGCTATCTGCCC, R-TAGAGCCTTCCTCTCCATCCG[25]

Nanog F-AGGGTCTGCTACTGAGATGCTCTG, R- CAACCACTGGTTTTTCTGCCACCG[27]

Oct4 F- GGCGTTCTCTTTGGAAAGGT, R-CTCGAACCACATCCTTCTCT[28]

Sox2 F-AGGGTTCTTGCTGGGTTTTGATT, R-CGGTCTTGCCAGTACTTGCTCTC[29]

Trp63 F-CAAAGAACGGCGATGGTACGAAG, R-GGCATGTGAGTGCCCATCATA[29]

Eya1 F- GGTGTGGAAGAAGAGCAAGGG, R-TGACACAAGAAGACAAGCCTGC[25]

FoxN1 F- ACTGACCTGGATGCTATCAACCC, R-TGTTTCTGCCAGACAAGGCC[25]

EpCAM F- GGGAGTCCCTGTTCCATTCTTC, R-CACCCATCTCCTTTATCTCAGC[25]

MHCII F-TCCGTCACAGGAGTCAGAAAGG, R-GCTGAGGTGGTGGATACAATAGTAC C[25]

### Quantitative PCR of TMSCs and sorted TECs

RNA was denatured in the presence of Random hexamers (Applied Biosystems N808-0127) and dNTPs at 65^O^C for 5 minutes. The denatured RNA was then used to synthesize cDNA using 5X buffer (Invitrogen y02321), DTT (Invitrogen y00147), SuperScriptIII (Invitrogen 18080-044), and RNase inhibitor (Invitrogen 1077-019) at 25^O^C for 10 minutes followed by 50^O^C for 50 minutes and 85^O^C for 5 minutes. The synthesized cDNA was subsequently used to preform quantitative real-time PCR using TaqMan universal master mix (Applied Biosystems 4369016) using the following TaqMan assay probes obtained from Applied Biosciences: 18srRNA (#4333760), Foxn1 (Mm00433946 m1) EpCAM (Mm00493214m1) Nanog (Mm02019550 s1), Oct4 (Mm03053917 g1), Sox2 (Mm00488369 s1). Applied Biosystems instrument default cycling protocol was used to perform q-RT-PCR using a 7500 RT-PCR system. 

### TMSC Cell culture and clonal cell line development

Thymic stromal cells were harvested and sorted based on the surface markers. Sorted cells were plated on 96 well plates coated with Laminin/Entactin (50μg/mL) in thymic stromal culture medium, MEM alpha containing 10% fetal bovine serum, penicillin-streptomycin, gentamycin supplemented with recombinant human LIF (10ng/mL), recombinant mouse EGF (50 ng/mL) and recombinant bFGF (20 ng/mL) (Invitrogen) until near confluence. Cells were gradually expanded into 24 well plates, 6 well plates and 100mm plates. Following expansion, cells were cloned by limiting dilution. Cells were passaged every 7 days. 

### Differentiation Assays

For adipogenic differentiation, 3 x 10^4^ thymic stromal cells or clonal TMSC cell lines were plated/ well of 6 well plates containing keratinocyte serum free medium (Invitrogen) supplemented with 10% fetal bovine serum, 0.18 mM calcium chloride, penicillin-streptomycin, gentamycin and recombinant mouse EGF (10 ng/mL), in the presence or absence of 5μM PPARγ inhibitor PW9662 (Sigma Aldrich), for 2 weeks with the medium changed every 3-4 days. 

For osteogenic differentiation, 3 x 10^4^ thymic stromal cells or cell lines were plated/well in 6 well plates containing Human/Mouse StemXVivo Osteogenic/Adipogenic Base Media supplemented with Mouse StemXVivo Osteogenic Supplement (R&D systems) for 2 weeks, with medium changes every 3-4 days. 

For chondrogenic differentiation, 3 x 10^4^ thymic stromal cells or clonal cell lines were maintained as a pellet after gentle centrifugation in 15 mL Falcon tubes containing Human/Mouse StemXVivo Chondrogenic Base Media supplemented with Human/Mouse StemXVivo Chondrogenic Supplement (R&D systems) for 3 weeks with medium changes every 3-4 days. Confirmation of adipogenic, osteogenic and chondrogenic differentiation were performed by Oil red O staining, Alkaline phosphatase and Alizarin Red S staining, and Alcian blue staining, respectively. 

### Mixed thymic reaggregates and kidney capsule transplants

Fetal thymic tissue was harvested from E15.5 fetal C57BL/6J mice. Tissue was cleaned to remove excess blood and adhering non-thymic tissue and then lobes were torn using forceps and washed repeatedly using a Pasteur pipette in 1 x PBS pH 7.4 to remove excess thymocytes. Embryonic thymi were digested with Dispase (1 mg/mL) and Deoxyribonuclease I solution (1 μg/mL), (Stem Cell Technologies) at 37°C for 15 minutes with occasional gentle agitation with a glass Pasteur pipette. The resulting single cell suspension was washed with PBS and passed through 100μm strainer (BD Bioscience) to remove any remaining undigested tissue. Adult thymus was dissociated from C57BL/6J eGFP-expressing mice (Jax), as described above, and subsets of adult stromal cells were sorted to greater than 95% purity on a BD FACS ARIAII sorter. 2-5 x10^5^ dissociated fetal stroma were mixed with 1-2 x 10^4^ FACS purified adult eGFP-expressing stroma and centrifuged at 1000G on a microfuge. Residual media was aspirated and the cell pellets were vortexed briefly. The resulting slurry was transferred to the surface of a 0.8μm polycarbonate filter (Millipore) supported on a transwell plate above RPMI FTOC medium and cultured for 48hrs at 37°C in 5% CO_2_. The resulting reaggregates were photographed to confirm the presence of eGFP-expressing adult cells and then surgically transferred under the kidney capsule of nude mice and allowed to grow for 3 weeks prior to harvest and preparation of cryostat sections.

## Results

### Localization and Characterization of H2BGFP label-retaining cells in the postnatal thymus

The K5tTA;TetO-H2BGFP double transgenic model, developed by Tumbar et al. [20] was used to identify label-retaining epithelial stem cells in the thymus. Briefly, mice expressing the tetracycline controlled transactivator as well as the Herpes Simplex Viral protein, VP16, driven by the Keratin 5 (K5) promoter (K5tTA) were bred to mice containing a transgene consisting of the tetracycline response element (TRE) ahead of a CMV promoter controlling expression of a histone 2B-green fluorescence protein fusion protein (tetO-H2BGFP). In the absence of the tetracycline analog doxycycline (Dox), all of the K5-expressing cells should express H2BGFP. Feeding Dox then inactivates tTA subsequently turning off expression of H2BGFP. Rapidly cycling cells quickly lose H2BGFP expression, while the label-retaining stem cells continue to express H2BGFP, allowing their localization *in situ* as well as their characterization by FACS and subsequent culture.

**Figure 1:**
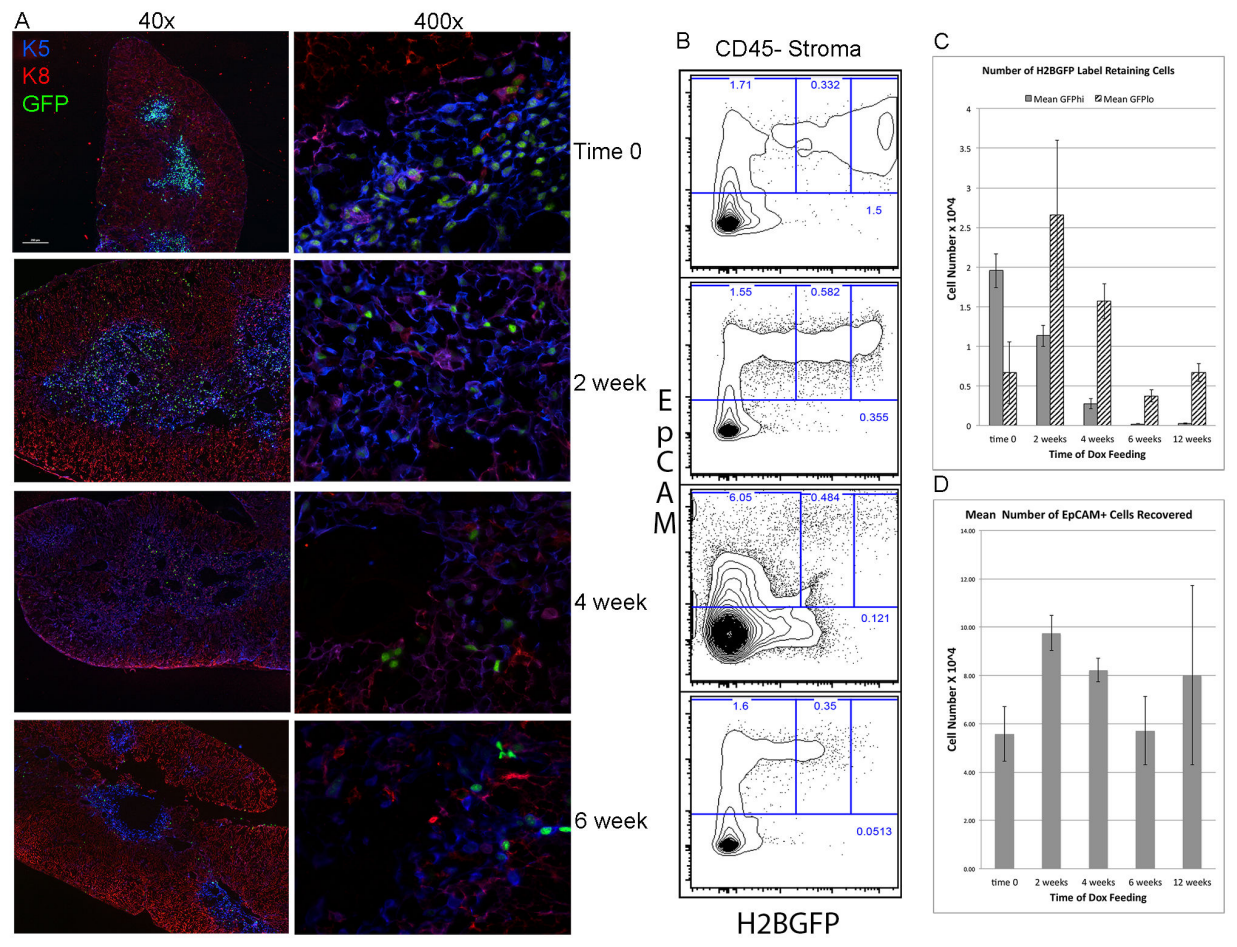
Changes in H2BGFP expression within TECs in K5tTA;TetO-H2BGFP transgenic mice following a time course of Dox feeding. A. Thymic sections prepared at the initiation of Dox feeding and from 2-6 weeks after the start of Dox feeding showing the reduction in H2BGFP expressing TECs. Sections were stained with anti-K8 (red) and anti-K5 antibodies (blue) to allow localization of the H2BGFP expressing nuclei within TECs. B. Representative FACS analysis of CD45^-^ dissociated thymic stroma showing the frequency of EpCAM^+^ H2BGFP^+^ cells at time 0, 2, 4 and 6 weeks after the initiation of Dox feeding to inhibit H2BGFP expression in K5^+^TECs. Gates from left to right in each panel show the frequency of EpCAM^+^H2BGFP^-^, EpCAM^+^H2BGFP^lo^ and EpCAM^+^GFP^hi^ TECs at each time point. C. Graph shows the mean number of EpCAM^+^H2BGFP^hi^ label-retaining cells and the number of EpCAM^+^H2BGFP^lo^ cycling cells per thymus at 0, 2, 4, 6 and 12 weeks after the inhibition of H2BGFP expression through Dox feeding. Error bars are +/- the standard deviation of the mean. Results are representative of 3 independent experiments with 5 mice at each time point/experiment. D. Graph depicts the mean number of CD45^-^ EpCAM^+^ TECs/thymus at 0, 2, 4, 6 and 12 weeks after the inhibition of H2BGFP expression through Dox feeding. Error bars are +/- the standard deviation of the mean.

To examine the kinetics of the H2BGFP reduction following the inhibition of H2BGFP expression by Dox, thymic sections were prepared at time 0, 2, 4, and 6 weeks after the initiation of Dox feeding and stained with antibodies to K5 and K8 to allow localization and characterization of the H2BGFP+ cells within the thymic architecture. K8 is expressed in the majority of cTECS, K5+K8+ TEC (proposed to contain TEC progenitors) [30-33], as well as a minority of more mature UEA1^hi^ mTECS. Prior to Dox feeding H2BGFP expression was strong in the K5^+^mTECs, as well as keratin 5-expressing cTECs at the cortico-medullary junction (CMJ) and scattered throughout the cortex and capsule of the thymus, as shown in the top panel of Figure 1A. Importantly, no expression was observed in either thymocytes or other stromal components. Our initial hypothesis was that if a thymic stem cell/progenitor existed, it might be localized in the band of K5^+^K8^+^ cells thought to contain the TEC progenitors that are known to expand during thymic regeneration [31,32]. Initial H2BGFP expression in those cells was a prerequisite for determining if LRCs were localized in that population. 

Following the induction of Dox feeding to inhibit H2BGFP expression within the K5+ TECs, a steady decline in H2BGFP expression was observed, characterized by both a loss of GFP intensity and a decrease in the frequency of GFP^+^ TECs (Figure 1 A, lower 3 panels). As early as 4-weeks after Dox was initiated, it was apparent that most of the H2BGFP^hi^ cells were localized to the CMJ at the border of the K8^+^ cTECs and the K5^+^mTECs and often within K5^+^K8^+^ TECs (see Figure 1A, 4 and 6 week 400X panels). While some GFP^hi^ cells remained scattered in the medulla at 4 weeks the majority mTECs deep in the medulla had reduced expression of GFP by 6 weeks. 

These histology results correlated very closely with the reduction in H2BGFP^+^ TECs observed when FACS analysis for EpCAM and GFP expression was performed on dissociated thymic tissue at each time point following Dox induction. EpCAM is the most definitive epithelial marker used to identify TECs [34]. GFP^hi^ cells represent the slow cycling label-retaining cells at later time points. GFP^low^ cells represent cycling cells in the process of diluting the label, while GFP^-^ cells represent TECs that either never expressed H2BGFP or cells that completely diluted the GFP due to rapid proliferation. At time 0 the frequency of EpCAM^+^GFP^hi^ TECs within the total CD45- stroma was 1.5% (Figure 1B); it then went down to 0.4% at 2 weeks, 0.12% at 4 weeks and 0.05% at 6 weeks. An expected increase in the GFP^low^ population that correlated with the dilution of H2BGFP expression in cycling cells was also observed. While the absolute frequencies varied across experiments due to inherent variation in the method used to dissociate the thymus, which results in a single cell suspension that is differentially contaminated with CD45^+^ hematopoietic cells, the relative changes were representative of 3 independent experiments. A comparison of actual numbers of EpCAM^+^GFP^hi^ cells over time, following Dox induction, showed a consistent reduction in GFP^hi^ cells from a high of ~2 x 10^4^/ thymus, representing 35% of the EpCAM^+^ TECs, reducing by 40-60% every 2 weeks to 0.2 x 10^3^/thymus at 6 weeks, representing 0.4% of the EpCAM^+^ TECs (Figure 1C, grey bars). A comparison of 6 and 12 weeks shows that the number of GFP^hi^ cells remains fairly constant, suggesting that they are truly LRCs. The number of GFP^lo^ cells increases initially and correlates inversely with the decrease in GFP^hi^ cells as expected (Figure 1C, striped bars). The number of EpCAM^+^ cells remains fairly constant ranging from 5.5-9.5 x 10^4^/thymus (Figure 1D). 

**Figure 2:**
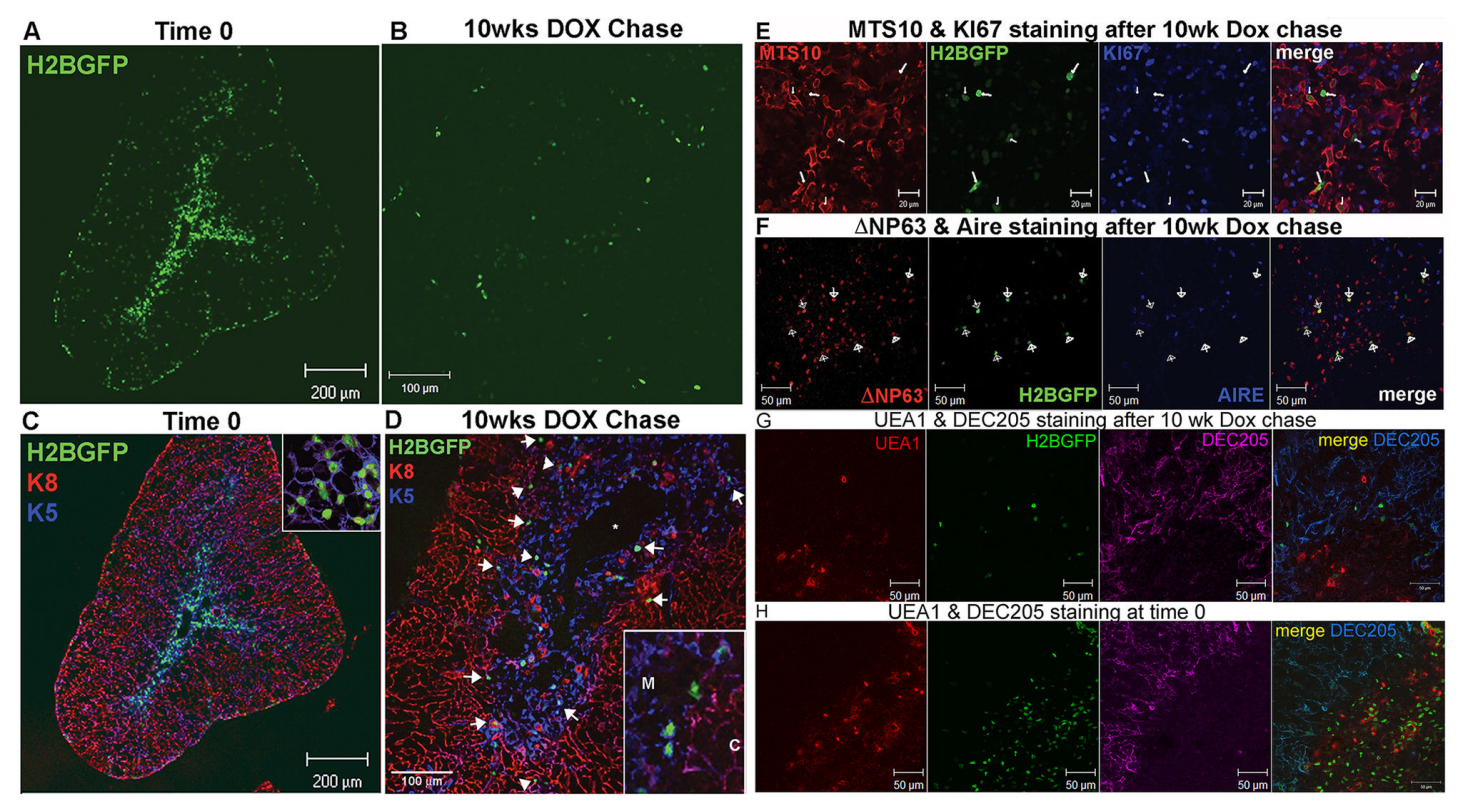
Localization and characterization of thymic label-retaining cells in K5tTA;TetO-H2BGFP mice. A. H2BGFP expression in thymic section prior to Dox feeding (100X). B. H2BGFP expression in thymic section after 10-week Dox feeding. C. Overlay of H2BGFP (green), K8 (red) and K5 (blue) expression prior to Dox feeding. White arrows show H2BGFP LRCs concentrated at cortico-medullary junction defined by K5 and K8 staining (inset= K5, H2BGFP overlay 400X showing that H2BGFP is restricted to K5-expressing cells). D. Overlay of H2BGFP, K8 and K5 in thymic section following 10-week Dox chase (inset: 400X of H2BGFP LRCs stained with K5 and K8 at CMJ). E. 400x images of thymic sections showing expression of MTS10, H2BGFP, Ki67 and merge. White arrows define position of H2BGFP^hi^ LRCs. F. 400X images of thymic sections showing expression of ΔNP63, H2BGFP, Aire and merge. White arrows define position of H2BGFP^hi^ LRCs. G 400x images of thymic sections showing staining with UEA1 PE (red) and DEC 205 Alexa 647 (pink) together with H2BGFP and Merge. H. 400x images of thymic sections derived from K5tTA;tetO-H2BGFP mice prior to Dox feeding stained with UEA1PE (red) and DEC205 Alexa 647(Pink) together with H2BGFP and merge.

Frozen sections of thymi from 3-week old K5tTA;TetO-H2BGFP double transgenic mice (H2BGFP mice) were prepared and stained for K5 and K8 expression. Prior to exposure to Dox, H2BGFP^+^ cells were abundant in both the cortex and medulla. H2BGFP expression was also evident in the capsular epithelium (Figure 2A). Counter staining with anti-K8 (red) and anti-K5 (blue) antibodies revealed that H2BGFP fusion protein expression was restricted to K5-expressing TECs found throughout the thymus. Importantly, no expression was observed in either thymocytes or other stromal components (Figure 2C and higher mag. inset). H2BGFP expression was more restricted to the medulla in 8-14 week-old mice (Figure 1A and 2H), reflecting the reduced expression of K5 in the cortex as the mice progress from neonates to adults. 

Dox feeding was initiated at weaning and continued for 10 weeks to allow dilution of the H2BGFP in the actively dividing TECs. Following the 10-week Dox chase analysis of H2BGFP expression in thymic sections revealed a dramatic reduction in the numbers of H2BGFP^+^ cells (Figure 2B). Counterstaining with K5 and K8 antibodies revealed that the H2BGFP^+^ label-retaining TECs were most abundant in a ring of cells localized at the cortico-medullary junction (Figure 2D). The white arrows and higher magnification inset identify H2BGFP^+^ cells at the junction between the K8-expressing (red) cortex and the K5-expressing (blue) medulla. In the higher magnification inset it is clear that the LRCs localized to the cortico-medullary junction maintain both K5 and K8 expression. 

All of the H2BGFP^hi^ LRCs localized within the edge of the medullary areas in thymic sections from Dox treated mice were positive for MTS10 antibody staining, which marks the dominant immature K5^+^ mTEC subset and typically surround clusters of more mature UEA1^hi^ CD80^hi^ Aire^+^ mTECs. K5^+^MTS10^+^ TECs define the first subset that can be identified as mTECs in the E12 thymus [30]. The LRCs did not express Ki67, which marks actively cycling cells (Figure 2E-white arrows indicate the location of H2BGFP^hi^ label-retaining cells), supporting that the LRCs had a stem cell phenotype similar to the slow cycling epithelial stem cells [20]. The LRCs in the thymus were also shown to express the N-terminal truncated isoform of P63, ΔNP63, but not the Aire transcription factor (Figure 2F). ΔNP63 is a P53 family member transcription factor expressed by epithelial stem cells in the skin and thought to define keratinocyte stem cells [32,35-38], and is lost as the cells differentiate [35,39]. P63 expression was also shown to be critical for thymic epithelial development, as P63KO mice exhibit a severely hypoplastic thymus [37]. The Aire transcription factor, on the other hand, is expressed by more mature mTECs (MHCII^hi^ CD80^hi^). The absence of Aire, which controls promiscuous gene expression necessary for self-tolerance, within the H2BGFP label-retaining subset argues against the label-retaining cells being terminally differentiated mTECs that have simply stopped dividing. This was confirmed when similar sections were stained with UEA1 (Ulex Europeus Agglutinin-1), which binds to most mTECs at a low level and strongly to MHCII^hi^ CD80^hi^ mature mTECs and anti-DEC205 (CD205), which labels all cTECs. Analysis of LRCs shows that they are primarily UEA1 low or negative when they are localized at the edge of the medulla while LRCs at the CMJ are primarily DEC205^+^ supporting a cTEC phenotype (Figure 2G). In contrast, when similar staining was performed on thymic sections derived from transgenic mice that had not been fed Dox, the GFP^+^ cells contained most of the mTECs including both UEA1^hi^ and UEA1^lo^ cells, as well as DEC205^+^ cells at the CMJ (Figure 2H) and K5^+^ cTEC scattered throughout the cortex (Figure 2A & C).

### Cell surface phenotype and in vitro growth potential of thymic label-retaining cells

The advantage of the H2BGFP transgenic model lies in the capacity to use flow cytometry to both analyze the cell surface phenotype of the label-retaining population and to sort viable LRCs for *in vitro* culture and gene expression analysis. To characterize the cell surface phenotype of the H2BGFP-expressing cells and follow the changes in that phenotype, FACS analysis was performed on dissociated thymic tissue both prior to the initiation of Dox feeding and at various times from 6-12 weeks following the inhibition of the H2BGFP-transgene by Dox. At each time point thymi from five mice were pooled and dissociated prior to staining with a panel of surface markers used to define TECs as well as stem cells. For each analysis residual CD45^+^ hematopoietic cells were excluded and then cells were gated for EpCAM expression to focus the analysis on TECs. The EpCAM^+^ cells were then analyzed for H2BGFP expression and gated for H2BGFP^hi^ versus H2BGFP^lo^ cells (Figure S1 A). For each subsequent analysis the surface expression observed at each time point in the Dox feeding time course is shown for the total CD45^-^ stroma, as well as for the total GFP^+^, GFP^lo^ and GFP^hi^ cells in each subsequent panel, respectively. The results shown are representative of 3 independent experiments.

Surface expression of MHCII and EpCAM is routinely used to analyze dissociated thymic stroma and allows characterization of cells into TEC and non-TEC subsets, with EpCAM defining the TECs and MHCII allowing further distinction of MHCII^hi^ mature mTECs and MHCII^lo^ immature mTECs and cTECs. MHCII^+^EpCAM^-^ cells represent a subset of thymic fibroblasts[12,40], while double negative cells include the remaining stromal components including MHCII^-^ fibroblasts and endothelial cells. Prior to Dox feeding the total GFP^+^ subset is restricted to primarily the EpCAM^hi^ TEC population and contains all of the MHCII-expressing TEC subsets including both the immature MHCII^lo^ and mature MHCII^hi^ subsets. The GFP^hi^ subset expresses a similar profile, however it is more enriched in the EpCAM^hi^MHCII^lo^ immature TEC subset (Figure S1 B). Following Dox feeding, the population of GFP^hi^ LRCs becomes progressively more restricted to the MHCII^lo^ subset (Figure S1B, lower right panel), suggesting that the cells are not likely to be terminally differentiated mTEC which fall in the MHCII^hi^ subset.

Further analysis with UEA1 and BP1 (Ly51), which are used to define mTECs and cTECs respectively, revealed an equal proportion of UEA1^+^ mTECs and BP1^+^ cTECs within the total GFP^+^ and GFP^hi^ subsets prior to Dox feeding. The GFP^hi^ LRCs gradually changed to a population that was more enriched in BP1^+^ cTECs with a small population of UEA1^+^ mTECs following 6 weeks of Dox feeding (Figure S1 C). These results correlated well with our histology data showing that the H2BGFP bright cells were primarily restricted to the CMJ within UEA1^dim^ immature mTECs and DEC205^+^ K5^+^K8^+^ mTECs. Analysis of MHCII together with CD80 revealed a similar trend, with the initial GFP^+^ and GFP^hi^ cells expressing a diverse TEC phenotype including both CD80^+^ mature mTECs and CD80^-^ immature mTECs and cTECs with the LRCs gradually showing a reduction in MHCII^hi^ CD80^hi^ cells but still containing 20% CD80^+^ cells at the end of 6 weeks of Dox feeding (Figure S1 D). These CD80^+^ cells could represent terminally differentiated mTECs that retained the label because they stopped dividing however, their lower MHCII expression level and slightly reduced CD80 expression more likely support an immature mTEC or cTEC identity.

**Figure 3:**
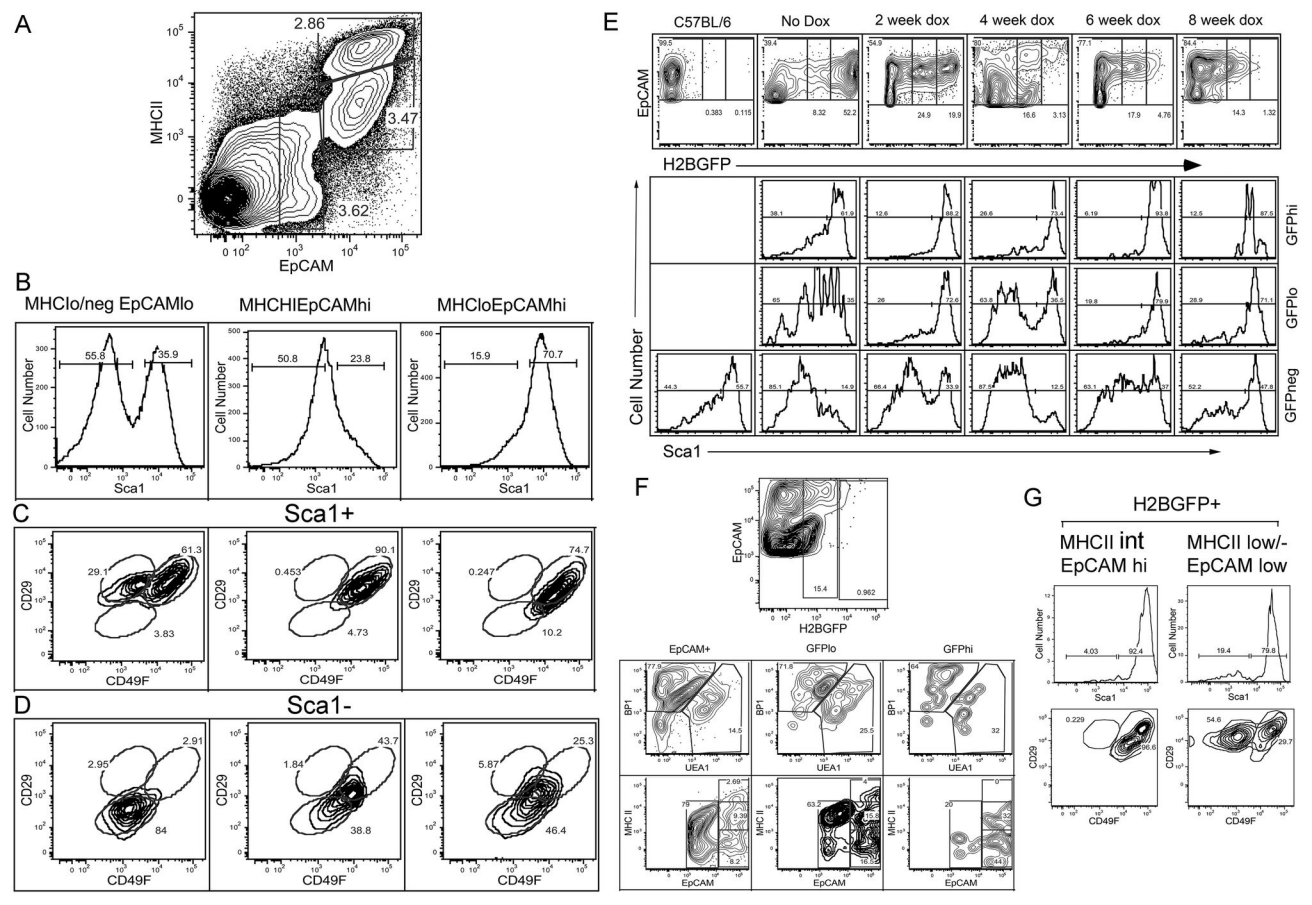
LRCs become progressively enriched in Sca1+TECs and contain a unique subset of MHCII^lo^EpCAM^lo^Sca1^+^CD49F^lo^CD29^+^ cells. A. Gating strategy for analysis of TEC subsets defined by MHCII and EpCAM expression in C57BL/6J dissociated thymic tissue. B. Sca1 expression within the 3 subsets defined by MHCII and EpCAM expression. C. Characterization of CD49F and CD29 expression within the Sca1^+^ TECs defined by MHCII and EpCAM expression. The MHCII^lo^EpCAM^lo^ Sca1^+^ TECs contain a unique CD49F^lo^CD29^+^ subset not found in other populations defined by MHCII and EpCAM expression or within Sca1^-^ TEC subsets. D. Characterization of CD49F and CD29 expression within the Sca1^-^ TECs defined by MHCII and EpCAM expression. E. Sca1 expression within the subsets of LRCs during an 8-week Dox time course. F. Characterization of EpCAM+ LRCs following 10-week Dox feeding. Upper panel shows gating used to define H2BGFP^hi^ and H2BGFP^lo^ subsets of LRCs. Middle 3 panels show characterization of total EpCAM+ as well as H2BGFPlo and H2BGFPhi LRCs using BP1 to define cTECs and UEA1 to define mTECs. Lower 3 panels show expression of MHCII and EpCAM in the same 3 populations. G. H2BGFP+ LRCs were separated into MHCII^lo^EpCAM^hi^ and MHCII^lo/-^ EPCAM^lo^ subsets and then analyzed for Sca1 expression (upper row). Sca1^+^ cells in each population were then analyzed for CD29 and CD49F. Results were representative of 5 independent experiments.

Stem cell antigen (Sca1/Ly6) is the most recognized hematopoietic stem cell (HSC) marker in mice [41-45]. Sca1 has also been identified in a number of non-hematopoietic tissues [46], however, and can be used to enrich for progenitor populations other then HSCs including epithelial stem cells [20,47] and MSCs [16]. Antibodies against Sca1, together with antibodies against the alpha-6 (CD49F) and beta-1 (CD29) integrins, have been used in combination to define MSCs and epithelial stem cells within the stem cell niche. Analysis of Sca1 expression on dissociated thymic stroma from a 3-month-old mouse revealed a surprising abundance of Sca1 expression on a variety of TEC subsets (Figure 3). After gating for CD45^-^ stroma, analysis with MHCII and EpCAM allowed separation of the EpCAM-expressing TEC subsets including the MHCII^hi^EpCAM^hi^ mature mTECs and the MHCII^lo^EpCAM^hi^ subset containing both immature mTECs and cTECs. The population of MHCII^lo/neg^EpCAM^lo^ TECs that is more abundant in older animals and appears in mice fed Dox for longer time periods was also analyzed (Figure 3A). The MHCII^hi^EpCAM^hi^ mature TEC subset was primarily Sca1^-^, while the less mature MHCII^lo^EpCAM^hi^ subset, which contains the LRCs, was predominantly Sca1^+^. The MHCII^lo/neg^EpCAM^lo^ TEC subset contained a mixture of 35-40% Sca1^+^ and 60-70% Sca1^-^ TECs (Figure 3B). No expression of Sca1 was detected in the MHCII^-^EpCAM^-^ stromal cells suggesting that Sca1 expression is restricted to EpCAM-expressing TECs in the CD45^-^ thymic stroma (data not shown). Analysis of CD49F and CD29 expression on the Sca1^+^ population derived from each TEC subset revealed that both the MHCII^hi^EpCAM^hi^ and MHCII^lo^EpCAM^hi^ TEC populations contained primarily CD49F^hi^CD29^+^ cells, while the MHCII^lo/neg^EpCAM^lo^ subset contained both a CD49F^hi^CD29^+^ subset and a CD49F^lo^CD29^+^ population (Figure 3C). In contrast the Sca1^-^ population in all TEC subsets contained primarily CD49F^lo^ CD29^-^ cells. These results were representative of three independent experiments. 

We next sought to determine if the H2BGFP^+^ LRCs expressed Sca1, similar to stem cell/progenitor populations in other tissues. Following gating for CD45^-^ EpCAM^+^ stroma the H2BGFP^-^, H2BGFP^lo^ and H2BGFP^hi^ subsets derived from sets of five mice, isolated before and up to 8 weeks after the initiation of Dox feeding, were analyzed for Sca1 expression (Figure 3E). With increased time after Dox induction, both the GFP^hi^ and GFP^lo^ subsets became progressively enriched in Sca1^+^ TECs, with the GFP^hi^ population being exclusively Sca1^+^ cells. The presence of Sca1 on the LRC subset supports an immature stem cell/progenitor phenotype. With the increase in age inherent in the K5tTA; tetO-H2BGFP mice, fed Dox for more than 8 weeks to ensure the dilution of the H2BGFP label, an increase in the MHCII^lo^EpCAM^lo^ TEC subset was observed including GFP^+^ cells. Analysis of GFP-expressing TECs following a 10-week Dox inhibition revealed the appearance of increased numbers of the MHCII^lo/neg^ EpCAM^lo^ subset, particularly in the GFP^lo^ subset (Figure 3F). This population was not abundant in the GFP-expressing cells in younger mice fed Dox for 6 weeks or less, suggesting that the cells are derived from GFP^hi^EpCAM^hi^ cells. Analysis of the H2BGFP^+^ LRCs within the MHCII^lo^ EpCAM^hi^ and MHCII^lo/-^EpCAM^lo^ subsets for expression of Sca1 showed that after 10 weeks of Dox feeding, both populations were enriched in Sca1^+^ cells (Figure 3G, upper 2 panels). Further analysis of the Sca1^+^ subsets for CD49F and CD29 expression revealed that the MHCII^lo/-^ EpCAM^lo^ Sca1^+^ population was enriched in a the unique CD49F^lo^CD29^+^ subset (Figure 3G lower 2 panels) which we will later demonstrate contains in vitro growth potential and the capacity to generate cell lines with characteristics of MSCs.

E12-E15 fetal epithelial cells contain a TEC progenitor phenotype and are able to form reconstitution thymic organ cultures (RTOCs), which are able to form functional thymic tissue when transplanted and grown under the kidney capsule of nude or syngeneic mice [48,49]. H2BGFP label-retaining epithelial stem cells, isolated from the skin, are able to form new hair follicles and skin [20]. To determine if LRCs sorted from the adult mouse thymus contained a population of TEC progenitors, 10,000 EpCAM^+^H2BGFP^+^ LRCs were sorted from 10-week Dox fed double transgenic mice and reaggregated with 2x10^5^ E15.5 dissociated fetal stroma and 2 x10^5^ sorted DN thymocytes. Removal from Dox treatment allows re-expression of the H2BGFP transgene and facilitates identification of the adult derived cells in the reaggregate. After 2 days in culture on transwell plates the reaggregates containing H2BGFP+ adult cells were transplanted under the kidney capsule of nude mice and allowed to grow for 4 weeks. While in all cases functional thymic tissue was present on the kidney due to the presence of fetal epithelial cells, significant numbers of H2BGFP expressing cells were only detected in 1 out of 12 reaggregates (data not shown) suggesting that they either do not contain a TEC progenitor population or that the adult stem cells are not able to compete or do not have the right environment to expand when placed in the fetal environment. We were encouraged, however, because in the one reaggregate that contained significant H2BGFP^+^ TECs the cells had clearly expanded and were expressing both cortical and medullary TEC markers. We chose not to present the results here because of the difficulty in reproducing the result, but it justifies further characterization of the LRC population. Forming reaggregates with only sorted adult cells was not possible due to the rare nature of the LRCs following Dox treatment and the large number of TECs required to consistently form reaggregates. 

**Figure 4:**
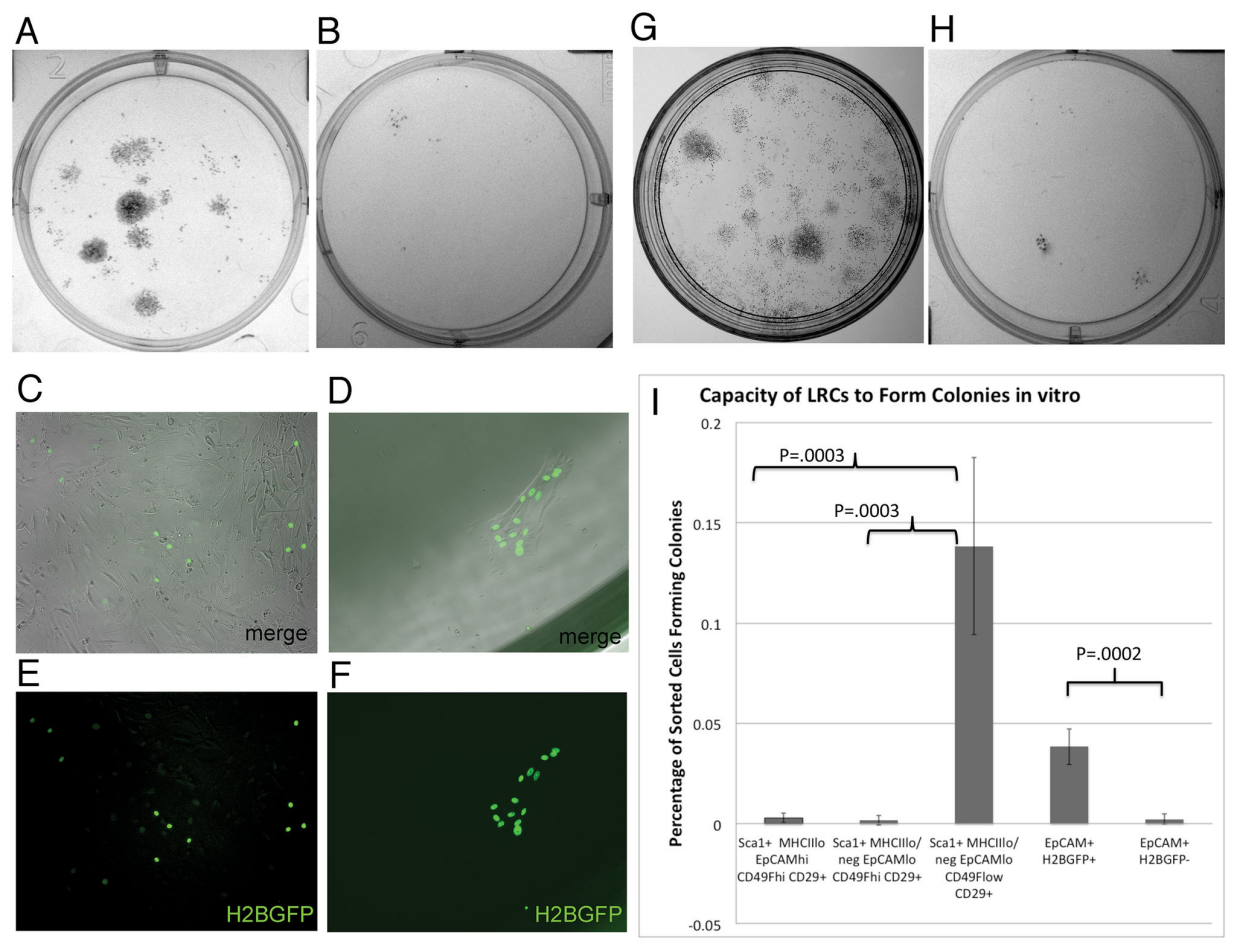
*In vitro* growth potential of H2BGFP LRCs. Methylene blue stained colonies derived from FACS sorted CD45^-^ EpCAM^lo^ MHCII^lo/-^Sca1^+^H2BGFP+ LRCs (A) or CD45^-^MHCII^int^EpCAM^hi^ Sca1^+^ H2BGFP^+^ stroma (B). C. Merge of H2BGFP expression and phase contrast image of expanded EpCAM^lo^MHCII^lo/-^ H2BGFP^+^ LRCs 1 week after sorting, demonstrating dramatic *in* *vitro* growth. D. Merge of H2BGFP expression and phase contrast image showing limited expansion of EpCAM^hi^ MHCII^int^H2BGFP^+^ LRCs 1 week after sorting. E. H2BGFP expression in same field as C. F. H2BGFP expression in same field as D. These results are representative of 3 independent experiments performed with 10-12 week Dox fed H2BGFP mice. Methylene blue stained colonies derived from FACS sorted CD45^-^MHCII^lo/-^EpCAM^lo^ Sca1^+^CD49F^lo^ CD29^+^ (G) or CD45^-^MHCII^int^EpCAM^hi^Sca1^+^CD49F^hi^CD29^+^ stroma (H) derived from WT C57BL/6J mice. I. Colony forming potential of sorted LRCs and defined TEC subsets sorted from dissociated thymus derived from postnatal C57BL/6J mice. Error bars show standard deviation of means calculated from 5 independent experiments. P values are derived by comparison of colony forming potential of sets of populations using T test.

Epithelial stem cells, as well as mesenchymal stem cells isolated from other tissues, share the capacity to grow and form colonies in culture [16,20,50,51]. When CD45^-^ EpCAM^lo^ MHCII^lo^ Sca1^+^H2BGFP^+^ LRCs were sorted to greater than 95% purity from dissociated 10-week Dox fed thymic tissue, and plated at a density of 3 x 10^4^ cells/plate they were shown to have a limited capacity to form colonies and grow when cultured in thymic stromal medium in the presence of LIF, EGF and FGF (Figure 4A). Counting the number of colonies formed revealed that the mean frequency of cells capable of forming clones was 0.04% (Figure 4I). In contrast, Sca1^+^H2BGFP^+^ CD45^-^EpCAM^hi^MHCII^lo^ cells, which are the dominant population of the GFP^hi^ LRCs, exhibited significantly less growth potential (0.002% mean, P=0.0002) under the same conditions (Figure 4B). Limited or no growth potential was also observed with sorted total EpCAM+ Sca1^-^, and EpCAM^hi^MHCII^hi^ Sca1^+^ (data not shown). 

We next compared the ability of the two subsets of Sca1^+^ thymic label-retaining cells to continue to grow in culture and generate cell lines. Sorted CD45^-^ EpCAM^lo^ MHCII^lo^ Sca1^+^ H2BGFP^+^ LRCs exhibited rapid *in vitro* growth when cultured in thymic stromal medium. The EpCAM^lo^ LRCs had reached full confluence within 4 days after sorting, when plated at 5 x 10^3^ cells/well in 96 well plates coated with laminin, with many cells maintaining H2BGFP expression (Figure 4C & E). In contrast, sorted MHCII^int^ EpCAM^hi^ Sca1^+^LRCs survived in culture and maintained stronger H2BGFP expression but only divided 1 or 2 times before halting their growth under identical conditions (Figure 4C & D). The reduction in H2BGFP expression continued in the EpCAM^lo^ cells until it was virtually undetectable under the fluorescence microscope at later passages. The morphology of the cells appeared similar to mesenchymal cells. Since the LRCs cells were derived from a Tet^off^ double transgenic mouse, subsequent culture in growth medium deficient in Dox allows for re-expression of H2BGFP driven by the K5 promoter. The expression of H2BGFP in the rapidly expanding population further confirms the epithelial origin of the LRCs, since H2BGFP expression in K5tTA;TetO-H2BGFP double transgenic mice was restricted to K5-expressing TECs (Figure 1). The reduction in GFP expression observed following longer culture and cloning could be the result of loss of epithelial characteristics and reduced K5-promoter activity. 

 Analysis of the MHCII^int^EpCAM^hi^ and MHCII^lo/-^EpCAM^lo^ GFP^+^ subsets for Sca1, CD49F and CD29 expression, following 10-weeks of Dox feeding, revealed that while both populations were predominantly Sca1^+^, they differed in their expression of CD49F. After gating for Sca1^+^ cells, both the EpCAM^hi^ and EpCAM^lo^ GFP^+^ subsets contained a population expressing high levels of both CD49F and CD29, while more than 50% of the MHCII^lo/-^ EpCAM^lo^Sca1^+^ population exhibited a CD49F^lo^CD29^+^ phenotype (Figure 3G). We reasoned that this unique population might explain the enhanced *in vitro* growth of the MHCII^lo/-^EpCAM^lo^ Sca1^+^ LRC subset. Sorted MHCII^lo/-^EpCAM^lo^ Sca1^+^CD49F^lo^CD29^+^ exhibited enhanced colony forming potential (Figure 4G) with a mean colony forming frequency of 0.14% +/- 0.04% SD (Figure 4I). In contrast, almost no growth was observed in either the MHCII^lo/-^EpCAM^lo^ Sca1^+^CD49F^hi^CD29^+^ or MHCII^int^EpCAM^hi^ Sca1^+^CD49F^hi^ CD29^+^ populations (Figure 4H & I). Together these results demonstrate that the label-retaining TEC subset with growth potential has a MHCII^lo/-^EpCAM^lo^ Sca1+CD49f^lo^CD29^+^ phenotype. Low-level expression of MHCII would be expected for more immature TECs, while the expression of Sca1, CD49F and CD29 is similar to the phenotype of label-retaining stem cells identified in the skin [20]. 

### LRCs isolated from the postnatal thymus maintain low-level H2BGFP expression and exhibit a cell surface phenotype similar to mesenchymal stem cells

**Figure 5:**
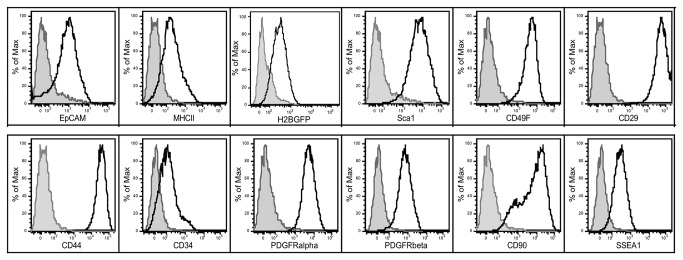
Culture expanded CD45^-^ MHCII^lo/-^EpCAM^lo^ Sca1^+^ CD49F^lo^CD29^+^H2BGFP LRCs maintain a surface phenotype similar to mesenchymal stem cells. After expanding sorted EpCAM^lo^ LRCs for 5 passages *in* *vitro*, their cell surface profile for a panel of TEC and mesenchymal stem cell surface proteins using flow cytometry. Overlay histograms show a comparison of staining for each antibody including EpCAM, MHCII, H2BGFP, Sca1, CD49F CD29, CD44, CD34, PDGFRα PDGFRβ, CD90 and SSEA in open histograms with the relevant isotype control antibody conjugated to the same fluorochrome in gray shaded histograms. The negative control for H2BGFP expression was TMSCs derived from C57BL/6J mice.

The fast growth rate and morphology suggested that the LRCs able to grow *in vitro* were similar to MSCs. We next sought to determine if the LRCs also shared molecular and functional characteristics of MSCs. After expanding the freshly sorted EpCAM^lo^ thymic LRCs for five passages in thymic stromal medium, one of these lines TMSC7 was stained with a panel of antibodies that define the cell surface profile of MSCs. This analysis revealed that the cells maintained low-level expression of H2BGFP, showing they were derived from LRC that had expressed keratin 5. H2BGFP expression is also confirmed in the fluorescence images shown in Figure 4. TMSC7 also maintained low-level surface expression of EpCAM, the most definitive marker of thymic epithelial cells, as well as low level MHCII expression. In addition, the culture expanded thymic LRCs maintained strong and consistent surface expression of Sca1, CD29, CD44, CD49F, CD90 and PDGFRα Low-level expression of CD34, PDGFRβ and SSEA was also detected. (Figure 5). TMSC lines also consistently expressed CD106 (data not shown). This surface profile is very similar to the surface profile of MSC lines derived from bone marrow and a variety of other tissues [16,52]. 

### EpCAM^lo^ Thymic LRCs expanded in vitro exhibit the capacity to differentiate into osteoblasts, adipocytes and chondrocytes similar to MSCs

**Figure 6:**
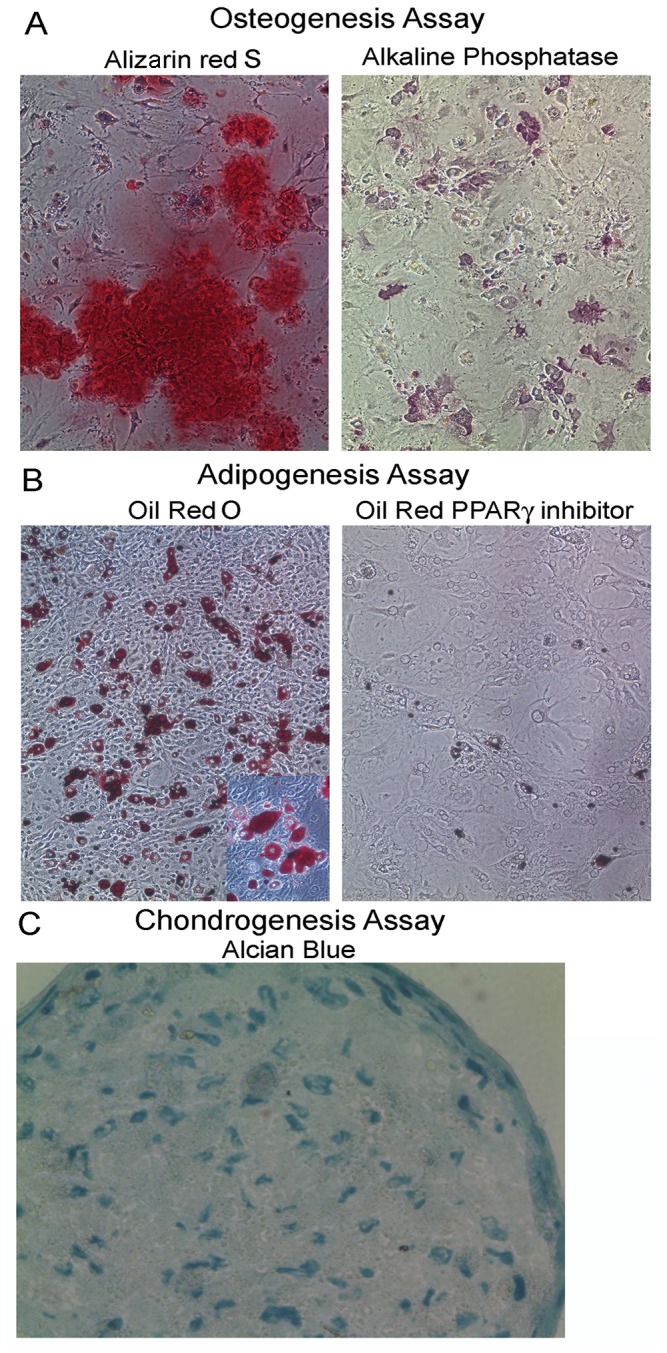
H2BGFP+ LRCs cultured *in vitro* exhibit the capacity to differentiate into adipocytes, osteoblasts and chondrocytes. A. (Left panel), Alizarin red staining of mineral deposits in culture-expanded FACS sorted CD45^-^Sca1^+^EpCAM^lo^ MHCII^lo^H2BGFP LRCs following 2-weeks in conditions that promote Osteogenesis; (Right panel), alkaline phosphatase activity characteristic of osteocytes, detected with DAB. B. Abundant oil red+ cells following 2 weeks of culture in adipogenesis conditions (inset shows 400X image of Oil red stained lipid deposits). C. Section of chondro-nodule stained with Alcian blue to detect mucin secreted by chondrocytes.

Based on the similarities to MSCs isolated from other tissues, we next investigated whether culture expanded sorted EpCAM^lo^ LRCs displayed MSC-like multipotency. MSCs derived from a variety of tissues are routinely characterized by their ability to differentiate into a number of distinct lineages including osteoblasts, adipocytes and chondrocytes [16,52]. After two weeks under culture conditions that promote osteogenesis, culture expanded EpCAM^lo^ LRCs displayed abundant mineral deposits, detected with alizarin red S staining, indicative of osteogenesis (Figure 6A, left). The cultures showed high alkaline phosphatase activity, also characteristic of osteoblasts [52] (Figure 6A, right). Culture expanded EpCAM^lo^ LRCs maintained in medium that allowed for *in vitro* growth showed no evidence of alizarin red S staining mineral deposits or alkaline phosphatase activity (data not shown). When cultured for two weeks in adipogenic conditions, very limited differentiation of adipocytes was observed. However, when cultured in Keratinocyte Serum Free Medium with low calcium and EGF, cultured EpCAM^lo^ LRCs differentiated into adipocytes indicated by the abundance of lipid deposition detected with Oil Red O (Figure 6B, left). When similar cultures were performed in the presence of PW9662, a PPARγ inhibitor known to limit adipogenesis, the number of oil red O positive adipocytes was dramatically reduced (Figure 6B, right). Finally, when EpCAM^lo^ LRCs were grown for three weeks in chondrogenic differentiation medium, chondro-nodules containing mucin-secreting cells detected by Alcian blue staining were observed. These data show that the cells maintain the capacity to differentiate into chondrocytes (Figure 6C). 

Taken together, the results presented thus far suggest that EpCAM^lo^ LRCs isolated from the postnatal thymus express a phenotype that is very similar to MSCs, including their cell surface phenotype, ability to expand in culture and their ability to differentiate into multiple lineages *in vitro*. While not a clonal cell line at this point in the analysis, the uniform staining pattern observed for each surface marker together with the high frequency of the cells able to differentiate suggests that the only cells to grow *in vitro* express a uniform MSC phenotype. 

### Generation of Clonal Thymic Mesenchymal Stem Cell-like lines from postnatal WT mice

 We next sought to produce long-term clonal cell lines derived from WT mice utilizing the phenotype determined from analysis of the H2BGFP LRCs described above. Thymi from 8-week old to 6-month old C57BL/6J mice were dissociated and subjected to partial depletion of hematopoietic cells using CD45 magnetic beads. The resulting stroma enriched population was then stained with antibodies against CD45, MHCII and EpCAM as well as Sca1, CD49F and CD29, identical to the analysis shown in Figure 3A-D. After gating for CD45^-^ cells to exclude remaining hematopoietic derived populations, analysis for EpCAM and MHCII allows for distinction of the MHCII^lo/-^EpCAM^lo^ population that exhibited enhanced growth potential (Figure 4A). This subset was then gated for the Sca1^+^CD49F^lo^CD29^+^ subset. Sorted control populations included the Sca1^+^ MHCII^lo/-^ EpCAM^lo^ CD49F^hi^ CD29^+^ and the Sca1^+^MHCII^lo^ EpCAM^hi^ CD49F^hi^ CD29^+^ subsets. All subsets were sorted to greater than 94% purity. Only the MHCII^lo/-^EpCAM^lo^ Sca1^hi^CD49F^lo^CD29^hi^ showed significant *in vitro* growth potential and was able to generate long term clonal cell lines (Figure S2), supporting our observations with the LRCs sorted from H2BGFP mice. Postnatal MHCII^lo/-^EpCAM^lo^ Sca1^hi^CD49f^lo^CD29^hi^ thymic stromal cells, isolated from 8-week to 6-month old mice, exhibit *in vitro* growth potential and can be consistently used to generate thymic stromal lines resembling MSCs. 

### Gene Expression Profile of Thymic Mesenchymal Stem Cell-like Lines

 Seven independent clonal thymic stromal lines with properties of MSCs (TMSC) have been grown in culture for up to 25 passages. These cell lines have maintained many characteristics of stem cells, as well as expression of genes associated with TEC development. When maintained in MEMα with LIF, EGF, FGF and 10% FBS medium, these cell lines exhibit a mesenchymal-like morphology that becomes more pronounced with passage (Figure S2 A & B). FACS analysis of two of those lines (TMSC7-10 and TMSC2-1), performed after 10 passages for the presence of the TEC markers EpCAM and MHCII, together with a panel of surface markers typically expressed on MSCs, revealed biphasic low level expression of EpCAM in TMSC7-10 while only a small subset TMSC2-1 expressed EpCAM. Limited expression of MHCII was observed in either TMSC line. EpCAM is the most definitive surface marker of TECs and while its expression was variable in intensity among the clones tested we consistently observed a biphasic profile with a small number of cells expressing higher levels of EpCAM. MHCII expression is typically lost from TECs upon removal from the 3D microenvironment of the thymus. Analysis with MSC markers revealed expression of stem cell antigen-1 (Sca1), hematopoietic cell E-selectin/L-selectin ligand (H CELL/CD44), β1 integrin (CD29), α6 integrin (CD49F), Thy1 (CD90), platelet derived growth factor receptor α & β (PDGFRα& β, lacto-glycolipid expressed on murine embryonic stem cells (SSEA-1), together with the absence of CD34 (Figure S3). With the exception of the low-level biphasic expression of the TEC marker EpCAM, the cell surface profile of both TMSC clones are identical to that reported on MSCs isolated from other tissues [16,50,52]. 

 To further characterize the TMSC lines, a gene expression profile was performed using a panel of primers specific for stem cell markers and transcription factors, as well as for genes typically associated with TEC development and function. RT-PCR of RNA isolated at passage 5 from TMSC7 prior to limiting dilution cloning, revealed that TMSCs maintained expression of the core transcriptional regulators that have been reported to be active in pluripotent cells including nanog, octamer-binding transcription factor 4 (Oct4) and sex determining region Y-2 (Sox2) [53], as well as a number of genes associated with the maintenance of pluripotency or early endoderm development including genesis/forkhead box D3 (FoxD3) [54], leucine-rich repeat-containing G-protein coupled receptor 5 (Lgr5) [55], forkhead box protein A1 (FoxA1) [56], Cdx1 [57], stella/developmental pluripotency associated 3 (Dappa3) [58] and undifferentiated embryonic cell transcription factor 1 (UTF1) [59]. TMSC lines also expressed genes reported to be important for TEC development including the transcription factors eyes absent 1 (EYA1), paired box gene 9 (PAX9) and forkhead box protein N1 (FOXN1) [60,61]. In addition, TMSCs maintained expression of Notch ligands delta-like ligand 1 (DLL1), delta-like ligand 4 (DLL4) and jagged1 (Jg1), Wnts including Wnt4 and Wnt10b, as well as EpCAM and MHCII (Figure S3 B). These genes are more typically associated with TECs within the 3D thymic microenvironment. Together, this gene expression profile suggests a population of cells that are unusual in their co-expression of both stem cell markers and genes more typically associated with more mature TECs and may support a rather immature or pluripotent phenotype for TECs in general. 

 Following two rounds of limiting dilution cloning, quantitative PCR was performed for the TMSC7-10 line after 16 passages and compared with freshly sorted TEC subsets including the MHCII^lo^ EpCAM^lo^ Sca1^+^CD49F^lo^ CD29^+^ subset, from which TMSC lines have been consistently derived. Highly purified (>95%) sorted MHCII^lo^ EpCAM^lo^ Sca1+CD49F^lo^ CD29^+^ TECs exhibit very limited expression of the most definitive TEC markers Foxn1 and EpCAM; however, they exhibited a 2.5 and 6 fold increase in the stem cell markers Nanog and Sox2, respectively, possibly contributing to their capacity to grow in culture and generate cell lines. Surprisingly, TMSC7-10 showed low-level but dramatically reduced expression of the stem cell markers Nanog, Oct4 and Sox2 and no maintenance of the TEC markers Foxn1 and EpCAM after longer *in vitro* culture (Figure S3 C) suggesting that these stem cell genes may not be responsible for the maintenance of pluripotency in TMSC lines and that the culture conditions used to maintain the TMSCs result in the loss of TEC characteristics with time. All results were normalized to the MHCII^int^ EpCAM^hi^ subset known to contain both cTECs and immature mTECs. 

### Clonal TMSC lines maintain the capacity to differentiate into multiple mesenchymal derived lineages

**Figure 7:**
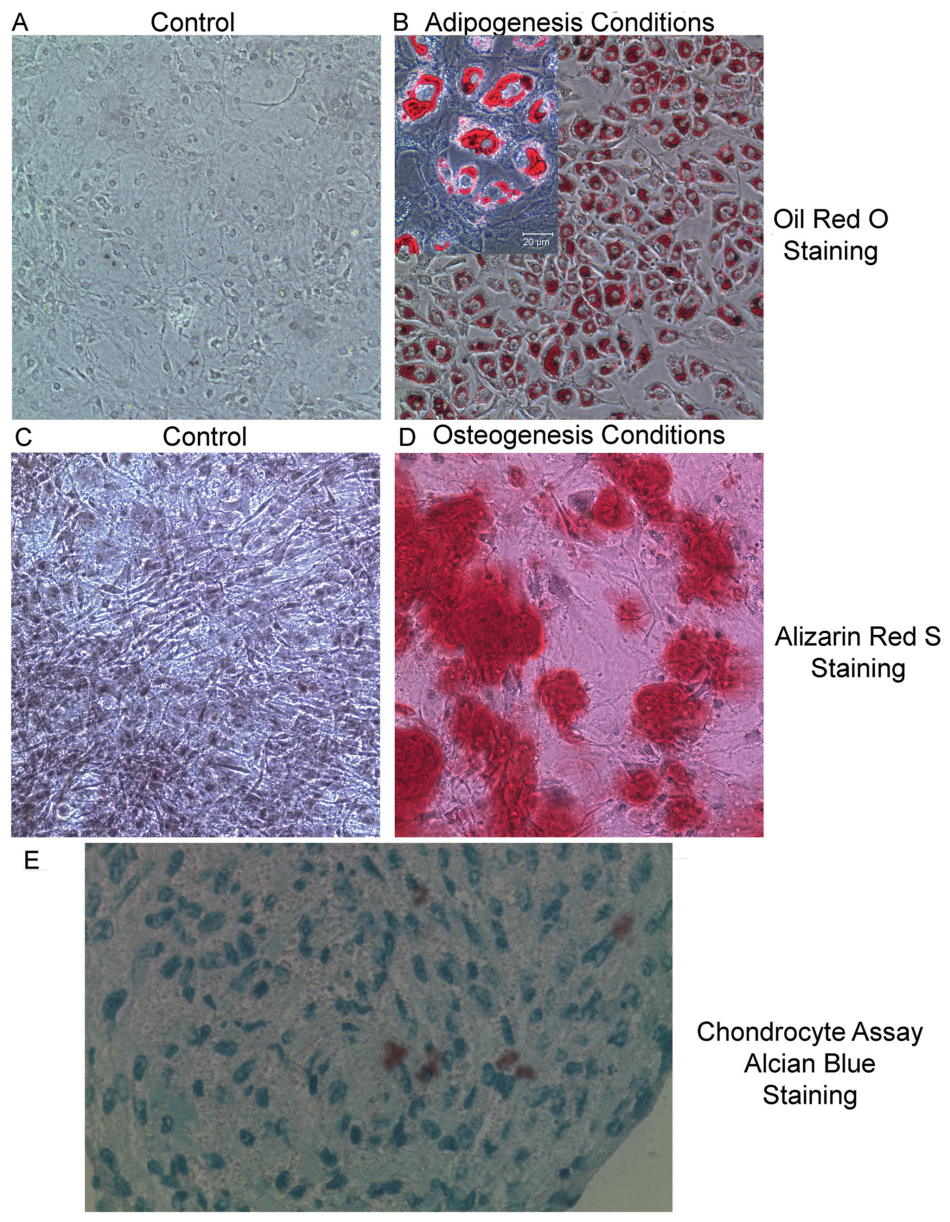
Clonal TMSC lines maintain the capacity to form Adipocytes, Osteoblasts and Chondrocytes *in vitro*. A. TMSC7 cell line cultured in MEMα medium supplemented with EGF, FGF and LIF and then stained with Oil Red O; B. Oil Red S staining of TMSC7 cultured in KSFM + 10% FBS + EGF which induced adipogenesis (inset 400X image of Oil Red O staining droplets of lipid); C. Alizarin Red S staining of TMSC7 cultured in control medium; D. Alizarin Red S staining of mineral deposits in TMSC7 cultured in osteogenesis differentiation medium; E. Alcian Blue staining of frozen section of Chondro-nodule following culture of TMSC7 cultured in chondrogenesis conditions. (Mag. A - D, 100X; inset and E 400X) Similar results for all differentiation assays were obtained in a minimum of 3 experiments and with multiple TMSC lines.

 The most definitive proof of MSC identity is the capacity to differentiate in to multiple Mesenchymal lineages under appropriate conditions. To determine if the TMSC lines, derived from EpCAM^lo^ MHCII^lo^ Sca1^+^CD49F^lo^CD29^hi^ thymic stromal cells sorted from 6-month old postnatal mice, maintained the capacity to differentiate into mesenchymal lineage cells, the TMSC7-10 line was subjected to the same culture conditions that promoted differentiation of EpCAM^lo^ MHCII^lo^ Sca1^+^H2BGFP LRCs (Figure 6). When subjected to conditions that induced adipogenesis in cultured thymic LRCs, TMSC7-10 differentiated into adipocytes as indicated by the presence of oil red O staining oil droplets within a large number of the cultured cells (Figure 7B and inset). TMSC7-10 control cultures grown in thymic stromal medium showed no evidence of oil red stained adipocytes (Figure 7A). When late passage cultures of TMSC7-10 cells are allowed to reach high density in thymic stromal medium a small number of cells appear to differentiate into adipocytes, suggesting a natural tendency to differentiate toward the adipocyte lineage, similar to aging TECs [9]. 

 Identical to cultured thymic LRCs, TMSC7-10 were also able to differentiate into osteocytes (Figure 7D) and chondrocytes (Figure 7E) when subjected to conditions that promote osteogenesis and chondrogenesis, respectively. Cultures of TMSC7-10 lines subjected to growth conditions that promote osteogenesis also stained for abundant expression of alkaline phosphatase, similar to the results shown for cultured LRCs in Figure 6 (data not shown). Identical results were obtained in 3 independent experiments and with 3 independent TMSC clones. Therefore, culture of thymic stromal cells defined by the EpCAM^lo^MHCII^lo^ Sca1^+^CD49f^lo^CD29^hi^ phenotype and identified as a subset of thymic LRCs, enriches for a population of postnatal thymic stromal cells with properties similar to MSCs. 

### Sorted EpCAM^lo^MHCII^lo^ Sca1^+^ TECs *used to derive TMSCs contribute to reaggregates with E15.5 fetal thymic stroma and are maintained after transplant under the nude mouse kidney capsule as TECs*

**Figure 8:**
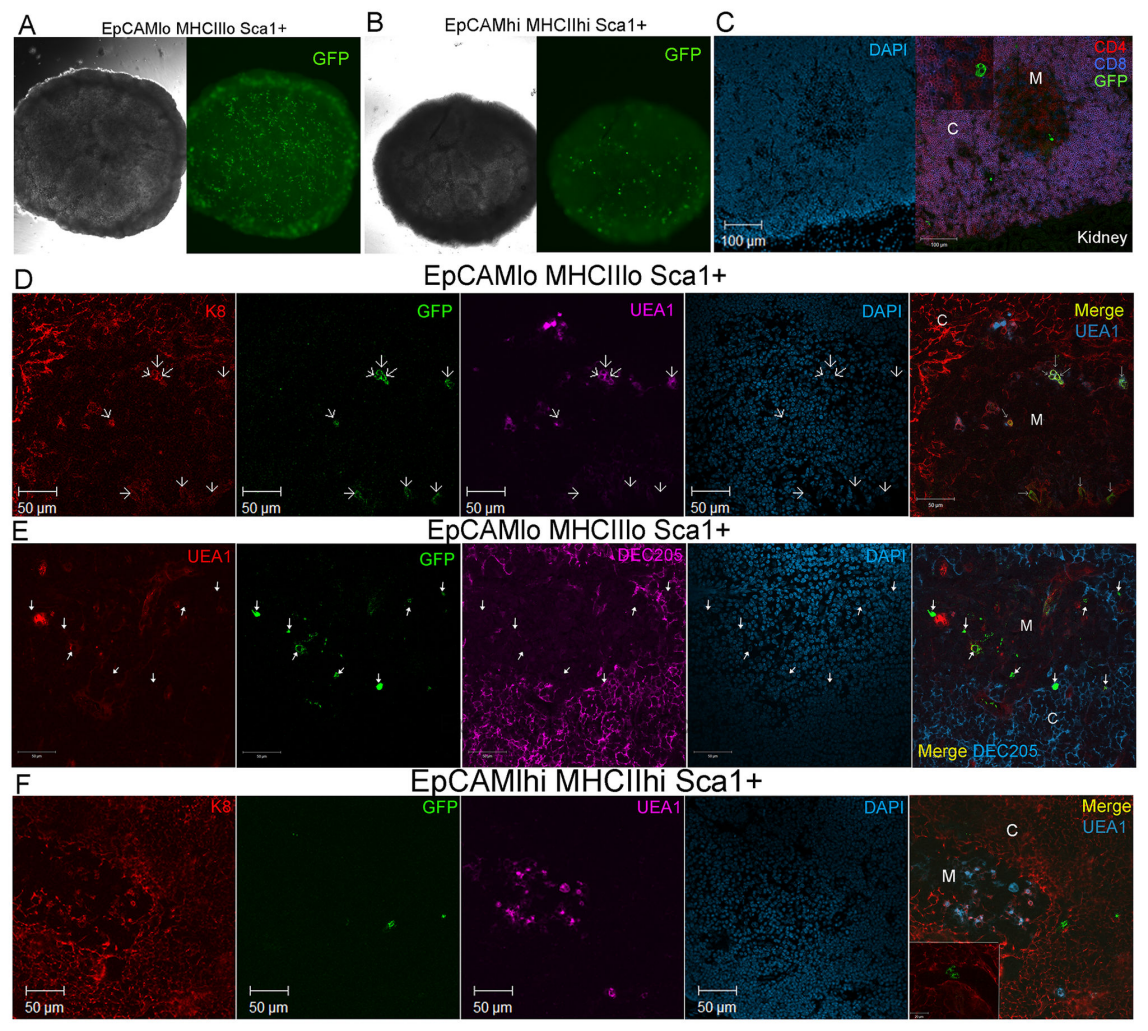
Sorted EpCAM^lo^ MHCII^lo^ Sca1^+^ TECs contribute to thymic reaggregates after transplant under the nude mouse kidney capsule. A. 40x phase and GFP fluorescence images of a reaggregate thymus created from sorted EpCAM^lo^MHCII^lo^Sca1^+^ TEC derived from B6eGFP mice mixed with dissociated B6 E15.5 fetal thymus. B. 40x phase (left) and GFP fluorescence images (right) of a reaggregate thymus created from sorted EpCAMl^hi^MHCII^hi^Sca1^+^ TEC derived from B6eGFP mice mixed with dissociated B6 E15.5 fetal thymus. C. 200X confocal fluorescence images of a 10μm frozen section prepared an EpCAM^lo^MHCII^lo^Sca1^+^ reaggregate thymus under the kidney capsule of a nude mouse stained with DAPI (left) and anti CD4PE (red), anti-CD8APC (blue) and anti-GFP FITC (green). The kidney is clearly visible on the lower right of each panel. D. Localization of sorted EpCAM^lo^ MHCII^lo^ Sca1^+^ GFP^+^ cells retained in sections of reaggregate thymus grown under nude mouse kidney capsule for 3 weeks. Arrows indicate the location of GFP^+^ cells in each panel. Sections were stained with anti Keratin 8-PE (red), antiGFP-FITC (green), UEA1 APC (pink) and DAPI (blue). Upper 5 cells were K8^+^UEA1^+^ while lower 3 only expressed K8. E. Sections of reaggregates containing EpCAM^lo^MHCII^lo^Sca1^+^ adult TECs stained with UEA1PE (red), Anti-GFP FITC (green) DEC205 Alexa647 (pink) and DAPI (blue). In the last merged panel GFP^+^ adult cells included UEA1^+^ and UEA1^-^ mTECs, as well DEC205^+^ cTECs. F. Localization of sorted EpCAM^hi^ MHCII^hi^ Sca1^+^ GFP^+^ cells retained in sections of reaggregate thymus grown under nude mouse kidney capsule for 3 weeks. Arrows indicate the location of GFP^+^ cells in each panel. Sections were stained with anti Keratin 8-PE (red), antiGFP-FITC (green), UEA1 APC (pink) and DAPI (blue). White C= cortex, White M= medulla in all panels.

The multipotency of TMSCs suggested that they might contribute to the maintenance of postnatal thymic epithelial microenvironments. To test this hypothesis, the EpCAM^lo^MHCII^lo^ Sca1^+^ thymic stromal subset, that consistently gave rise to TMSC lines *in vitro*, was sorted from C57BL/6J eGFP mice that ubiquitously express GFP in all cells. EpCAM^hi^MHCII^int^ Sca1^+^ and EpCAM^hi^MHCII^hi^ Sca1^+^ subsets with no *in vitro* growth potential were also sorted as controls. Cells were sorted to greater than 95% purity (data not shown). The sorted GFP-expressing subsets were mixed with dissociated thymocyte-deleted fetal stroma derived from E15.5 C57BL/6J WT mice and allowed to form reaggregates for 48hrs on polycarbonate filters supported on transwell plates. Functional reaggregates were photographed in phase and with 488nm excitation to show that the sorted eGFP adult stromal subsets were maintained in the reaggregates (Figure 8A-B). All of the sorted eGFP-expressing adult subsets were present within the reaggregate cultures at 48hrs, however the EpCAM^lo^MHCII^lo^ Sca1^+^ subset was more abundant and the cells appeared to have more cytoplasmic processes (Figure 8 A, right panel) when compared with control subsets like the EpCAM^lhi^MHCII^hi^ Sca1^+^ population (Figure 8B, right panel). These results were representative of three independent experiments performed with 2-3 reaggregates of each type for each experiment. Reaggregates attempted with multiple clonal GFP-expressing TMSC lines alone failed to make functional reaggregates with sorted DN thymocytes, possibly due to loss of TEC characteristics after prolonged culture. In reaggregates, generated by mixing fetal thymic stroma and limited numbers of GFP-expressing TMSC lines the GFP^+^ cells that remained were never found to co-express keratin suggesting that the cells may have lost the capacity to differentiate into TECs or that the fetal environment is not appropriate for their differentiation into TECs. 

Reaggregates containing sorted EpCAM^lo^MHCII^lo^ Sca1^+^ eGFP^+^ adult stromal cells were then surgically transplanted under the kidney capsule of nude mice. Following 3 weeks of growth under the kidney capsule, the kidneys were harvested, embedded, and cryostat sections were prepared. Sections were stained with anti-GFP to detect GFP expressing adult stromal cells maintained in the expanded reaggregate thymus, as well as a panel of antibodies used to characterize both thymocytes and the thymic stroma. 

To confirm that the reaggregate thymi observed growing under the kidney capsule were functional, sections were prepared and stained with anti-CD4 and anti-CD8 to confirm the presence of developing thymocytes recruited from the nude mouse bone marrow (BM), and to characterize the distribution and developmental state of the thymocytes present. When thymic sections are stained with DAPI, cortical areas have a higher density of nuclei due to the abundance of DP thymocytes, while medullary areas exhibit a lower density of staining due to the reduced number of mature CD4 and CD8 SP. This was clearly evident in all of the reaggregate thymi produced (Figure 8C, left panel). Staining with CD4 and CD8 confirmed the presence of abundant DP thymocytes (purple) in cortical areas as well as CD4SP (red) and CD8SP (blue) in medullary areas, confirming the capacity of the reaggregate thymus to attract T cell progenitors from the BM and support T cell development to the mature SP stage. GFP^+^ cells derived from the EpCAM^lo^MHCII^lo^Sca1^+^ cells were seen in both medullary and cortical areas and in some cases were found included in GFP-expressing clusters of cell that could represent expanding cells (Figure 8C, inset)

The higher abundance of GFP-expressing cells observed in the reaggregates after the 2 day *in vitro* culture (Figure 8A) was maintained in the reaggregates created with the EpCAM^lo^MHCII^lo^ Sca1^+^ subset, with the majority of the GFP-expressing cells being localized in the medulla or cortico-medullary junction (Figure 8D & E). While dramatic expansion of the GFP-expressing adult cells was not apparent, staining with anti-K8 (expressed in the majority of cTECs and a subset of mTECs) as well as the mTEC marker UEA1, demonstrated that the GFP-expressing cells remaining after 3 weeks had become both UEA1^+^K8^+^ TECs similar to mature mTECs (Figure 8D upper 4 arrows), as well as an equal or higher number of K8^+^UEA1^-^ cells, which might be cTECs or K5^+^K8^+^ putative TEC progenitors. Further characterization with the cTEC specific DEC205 antibody together with UEA1 revealed an abundance of GFP-expressing UEA1^lo/neg^ cells in the medulla (likely to be immature mTECs), as well as DEC205^+^ cTECs localized at the cortico-medullary junction. In contrast to these results observed in reaggregates made with the EpCAM^lo^MHCII^lo^ Sca1^+^subset, none of the limited number of GFP-expressing cells that were maintained in reaggregates created with sorted control populations expressed an epithelial phenotype (Figure 8F), as they did not express K8, DEC205, MTS10 or bind UEA1. Most of the GFP^+^ cells that remained appeared to be localized within epithelial free regions of the thymus or possibly lining or within blood vessels (Figure 8F and inset). The requirement for the use of the rabbit polyclonal anti-GFP antibody to detect the GFP-expressing cells limited our capacity to counter-stain with rabbit anti-K5 or anti-K14 antibodies, as no other anti-GFP antibodies or keratin antibodies were functional and specific in our hands. 

## Discussion

Utilizing the slow cycling characteristics typically associated with stem cells, this study set out to isolate a population of TECs with properties of stem cells from the postnatal thymus. The K5tTA;TetO-H2BGFP tetracycline-regulated transgenic model was utilized to facilitate the use of flow cytometry for characterization and subsequent sorting of LRCs for *in vitro* growth potential and gene expression. Prior to Dox induced inhibition of H2BGFP expression, immunofluorescence microscopy revealed that H2BGFP transgene expression was restricted to K5-expressing TECs and labeled the dominant K5^+^mTEC subset, as well as K5K8DP Dec205^+^ cTECs and capsular epithelium. Following a 6 to 12-week Dox chase, the expected reduction in H2BGFP expression was observed, leaving a small population of H2BGFP^hi^ LRCs at the cortico-medullary junction and H2BGFP^low^ LRCs primarily within the medulla. Analysis of these LRC populations by histology revealed that they expressed immature TECs markers (MTS10 and K5 or K5K8DP) as well as the N-terminal deleted isoform of P63 (ΔNP63). ΔNP63 identifies keratinocyte stem cells [36] and is essential for the proliferative potential of stem cells in stratified epithelia [37]. ΔNP63 is expressed in a large percentage of TECs and loss of P63 results in a severely hypoplastic thymus [37]. A larger population of H2BGFP^dim^ LRCs cells was also observed and persisted for at least 8 months when mice were fed Dox for an extended time. This population was most abundant in the K5-expressing thymic medulla, however it appeared to express a more diverse phenotype including some cells that had reduced keratin expression, suggestive of epithelial mesenchymal transition (EMT) (manuscript in preparation). Alternatively, the dim cells may represent cells that have been derived from the brighter LRCs that exhibit reduced H2BGFP expression due to a high rate of proliferation. 

Analysis of the thymic LRCs using FACS revealed a surprising abundance of H2BGFP^+^ LRCs and confirmed our histological observations with a small number H2BGFP^bright^ cells and a larger percentage of H2BGFP^dim^ cells, even after suppression of H2BGFP expression for eight months. Further analysis of the LRCs using EpCAM and MHCII expression, which define TECs, revealed two distinct subsets of LRCs including both EpCAM^hi^ MHCII^int^ and EpCAM^lo^ MHCII^lo^ subsets. The H2BGFP^hi^ subset contained primarily EpCAM^hi^ MHCII^int^ TECs while the H2BGFP^dim^ subset contained primarily EpCAM^lo^ MHCII^lo^ TEC subsets. Initial analysis of the growth potential of EpCAM^+^ LRCs compared with EpCAM^+^ non-label-retaining cells demonstrated enhanced *in vitro* growth of the LRCs. 

Culture of sorted populations of the EpCAM^lo^ versus the EpCAM^hi^ LRC subsets revealed that only the EpCAM^lo^ LRC subset exhibited robust *in vitro* growth, a property typically associated with stem cells. Initially these cells maintained bright H2BGFP expression (Figure 4) confirming that they were derived from K5-expressing label-retaining epithelial cells, since the transgene is driven by the K5 promoter and histology confirmed that the transgene was restricted to K5^+^ epithelial cells in the thymus (Figure 1 and 2). Cell surface analysis of this population following expansion *in vitro* revealed a cell surface profile virtually identical to MSCs while the cultured cells also maintained expression of H2BGFP (confirming their derivation from label retaining K5-expressing TECs) as well as both EpCAM and MHCII (Figure 5). Subsequently, differentiation assays performed with culture expanded LRCs demonstrated that, like MSCs, they maintained the ability to differentiate into multiple lineages including osteoblasts, adipocytes and chondrocytes (Figure 6). 

MSCs and tissue specific stem cells show a remarkable conservation of cell surface markers irrespective of their origin. Sca1 was shown to be present on hematopoietic, prostate, lung, cardiac and mammary stem cells, as well as MSCs isolated from numerous sources [16,44,47,62-65]. α6 integrin/CD49F also shows a wide distribution among stem cell types, especially when it is analyzed with its subunit pair integrin β1/ CD29. Enrichment for skin stem cells was achieved through sorting CD49F^hi^CD29^hi^ populations [66,67]. Stingl et al [68] demonstrated that mammary stem cells express a Sca1^lo^CD49F^hi^CD29^hi^ phenotype, while an identical phenotype was reported for murine prostate stem cells [65]. Microarray analysis has shown that CD49F is overexpressed consistently in neural, hematopoietic and embryonic stem cells [69]. Finally mesenchymal stem cells isolated from a variety of tissues and organs have also been shown to express Sca1, CD49F and CD29 as well as CD44 [16,70,71]. 

Using the cell surface phenotype identified for the H2BGFP LRCs with *in vitro* growth potential, EpCAM^lo^MHCII^lo^Sca1^hi^CD49^lo^CD29^hi^, multiple clonal TMSC lines were created by sorting the same population from up to eight-month old WT mice. Comparison of the FACS analysis of the LRCs versus the stroma derived from WT mice (Figure 3) revealed one significant difference that confirms that label retention does enrich for a stem cell population in the thymic stroma. When the EpCAM^lo^MHCII^lo^ subsets were compared for Sca1 expression, the WT stroma contains both a Sca1^lo^ and Sca1^hi^ subset (Figure 3B), while the LRCs contain primarily Sca1^hi^ cells (Figure 3E & G). When the Sca1 high and low subsets isolated from WT mice were further analyzed for expression of CD49F and CD29, only the Sca1^hi^ subset contains the CD49F^lo^CD29^hi^ subset, which exhibited properties of mesenchymal stem cells including *in vitro* growth potential and significant pluripotency. While not sufficient to completely identify the stem cell population, label retention enriched for a population of thymic stroma with stem cells properties, even when derived from an eight-month old postnatal thymus. This demonstrates that the postnatal thymus contains a population of pluripotent stem cells within the EpCAM^lo^MHCII^lo^Sca1^+^CD49F^lo^CD29^hi^ subset of thymic stromal cells. These cells maintain properties of TECs including EpCAM and FoxN1 that are routinely used to define thymic epithelium, together with a previously unrecognized pluripotency more commonly associated with MSCs or other stem cell populations. 

Like the culture-expanded LRCs, TMSC lines maintained a cell surface phenotype similar to MSCs and the ability to differentiate into multiple lineages including adipocytes, osteoblasts and chondrocytes when subjected to differentiation assays. Gene expression analysis of culture expanded LRCs revealed a surprisingly diverse gene expression pattern including a number of core transcription factors typically associated with pluripotent stem cell populations including Nanog, Oct4, Sox2, Dppa3 and Lgr5, while at the same time they expressed genes typically associated with TECs including EpCAM, MHCII and FoxN1, as well as Notch ligands Dll1, Dll4 and Jagged1. TECs are known to express a diverse set of peripheral genes, termed tissue restricted gene expression, driven by the Aire transcription factor [72]. Tissue-restricted gene expression is needed for deletion of T cells bearing self-reactive T cell antigen receptors that could ultimately mount autoimmune responses. Analysis of Aire expression by PCR and staining with anti-Aire antibodies showed that none of the TMSC lines expressed Aire, excluding the possibility that the diverse set of genes expressed by TMSCs represented Aire dependent tissue restricted antigens. Single cell analysis was not performed so it is possible that different subsets of cells existed after prolonged culture that expressed unique combinations of pluripotent and more mature TEC markers. However, differentiation assays and cell surface analysis performed on multiple cell lines at different passages gave consistent and repeatable results suggesting that the culture conditions used are maintaining a pluripotent phenotype within a population of cells that still maintain characteristics that mimic the epithelial microenvironment from which they were derived. 

Epithelial cells are known to arise from all three germ layers, although thymic epithelium has been shown to arise exclusively from the third pharyngeal pouch endoderm [73]. Epithelial cells are typically characterized by the expression of cytokeratins together with strong intercellular connections and cell polarity. Mesenchymal cells express vimentin intermediate filaments and lack distinct intercellular connections [74]. TECs exhibit a less defined polarity, express a variety of keratins and are further defined by expression of EpCAM. Typical undifferentiated MSCs do not express cytokeratins or other epithelial specific molecules such as occludin or E-cadherin, however, epithelial cells and mesenchymal cells are known to share a reciprocal plasticity. Through the processes of Epithelial Mesenchymal Transition (EMT) and Mesenchymal Epithelial Transition (MET), cells are thought to shift as needed between the two phenotypes during normal embryonic morphogenesis, tumor progression and tissue repair [74]. Indeed, EMT has been described in the thymus and may contribute to age associated thymic involution and the loss of functional TECs that are replaced by fibroblasts and eventually fat in the aging thymus [9-11]. This might explain the unusual phenotype of the TMSCs described in this study, which appear to share characteristics of both thymic epithelial cells (K5 driven H2BGFP, Foxn1, EpCAM and MHCII expression) and MSCs (cell surface phenotype, *in vitro* growth potential, morphology and multipotency). Analysis of long-term clonal TMSC lines showed that MHCII and Foxn1 are completely absent, while EpCAM expression is highly reduced, suggesting that culture selects against a TEC phenotype. Interestingly, mammary epithelial cells that were artificially induced to undergo EMT took on properties of MSCs including gene expression and potential for multipotency, much like the TMSCs that naturally occur in the postnatal thymus [75]. EMT has also been associated with the acquisition of stem cell like characteristics in tumor cells, which may be causally linked to tumor recurrence [70,76,77]. It is intriguing to speculate that EMT occurring within the thymus as a natural part of thymic aging may contribute to the development of multipotent stem cells. Alternatively, the changes in morphology and gene expression observed might reflect an inherent pluripotency of TECs coupled with their removal from the thymic microenvironment and expansion in culture. Recently, significant pluripotency of TECs was demonstrated when cell culture expanded TECs derived from fetal and newborn rat thymus where found to form colonies in culture and could be reprogrammed into skin multipotent stem cells when transplanted into mouse skin in a wound healing assay [78]. Microenvironment clues were sufficient to redirect epithelial fate allowing the cells, shown to maintain characteristics of epithelial cells *in vitro*, to cross primitive germ layer boundaries. It will be interesting to determine if this is a general property of thymic stromal components and analyze the potential of the TMSCs, described in this study, to respond to different *in vivo* microenvironments to determine if the postnatal thymus represents a source of multipotent or truly pluripotent stem cells. 

A number of studies have demonstrated that MSCs isolated from a variety of sources have the capacity to differentiate into mesodermal cell types including chondrocytes, osteoblasts, adipocytes and myocytes when subjected to growth in selective media with lineage specific inductive factors [16,79-86]. Surprisingly, MSCs also appear to have the capacity to differentiate into cells of both ectodermal and endodermal origin. A combination of *in vitro* and *in vivo* studies has documented differentiation of MSCs into neural cells, hepatocytes, pancreatic islet cells, endothelial cells and epithelial cells [87-96]. Future efforts will investigate whether the TMSCs identified in this study contribute to maintenance of postnatal thymic epithelial microenvironments. While freshly sorted EpCAM^lo^MHCII^lo^Sca1^+^CD29^+^CD49F^lo^ TECS are maintained in reaggregates and express epithelial characteristics after three weeks of growth under the nude mouse kidney capsule (Figure 8), GFP-expressing clonal TMSC lines are maintained in small numbers but do not differentiate into TEC under similar conditions (data not shown). Current efforts are aimed at identifying culture conditions capable of inducing a TEC progenitor phenotype in TMSC lines, similar to recent experiments performed with ES cells that were used to generate functional thymic epithelium [97]. 

Due to the avoidance of ethical concerns associated with embryonic stem cells and the technical challenges and risks associated with iPS technologies, adult stem cells are the preferred choice for use in regenerative medicine. This is particularly true for diseases like osteoporosis and osteoarthritis where adult tissue derived stem cells might be used to replace lost bone or cartilage. Thymic tissue is often removed as a by-product of human heart surgeries, so the ability to easily derive MSCs from thymic tissue could provide a potential source of human MSCs. Consistent and reproducible differentiation of multiple TMSC lines into osteoblasts and chondrocytes makes the thymus a viable alternative to bone marrow as a rich source of MSCs.

## Supporting Information

Figure S1 **Cell Surface Profile of H2BGFP LRCs.** A. Gating strategy for analysis of LRCs based on EpCAM and H2BGFP expression during 0-6 week Dox feeding time course. Gate frequencies from left to right show total H2BGFP^+^, H2BGFP^lo^ and H2BGFP^hi^, respectively. B. Characterization of changes EpCAM and MHCII expression in total CD45^-^ stroma, total GFP^+^, GFP^low^ and GFP^hi^ subsets, every 2 weeks during a 6-week Dox time course. C. Characterization of changes in the frequency of MHCII versus CD80 in total CD45^-^ stroma, total GFP^+^, GFP^low^ and GFP^hi^ subsets, every 2 weeks during a 6-week Dox time course. D. Characterization of changes EpCAM and MHCII expression in total CD45^-^ stroma, total GFP^+^, GFP^low^ and GFP^hi^ subsets, every 2 weeks during a 6-week Dox time course.
(TIF)

Figure S2 **Morphology of TMSC lines in culture.** A. Phase image of TMSC7-10 at P3; B. Phase image of TMSC2-1at P12.
(TIF)

Figure S3 **Gene Expression Profile of clonal TMSC lines.** A. Clonal TMSC lines exhibit a surface profile similar to mesenchymal stem cells.
Cell surface profile of TMSC7-10 and TMSC2-1 cell lines after 10 passages. For each antibody overlay, the grey filled histogram shows isotype control antibody staining and the solid and dotted black histograms shows staining with the specific antibody for the TMSC7-10 and TMSC2-1 cell lines, respectively.
B.. Rt-PCR analysis of RNA isolated from TMSC7. Rt-PCR analysis of RNA isolated at P7 from TMSC7 revealed expression of core transcription regulators of pluripotent cells 1) Nanog, 2) Oct4 3) Sox2; Genes involved in the maintenance of pluripotency 4) Foxd3, 5) Lgr5, 6) Dppa3, 7) Utf1; transcription factors involved in early development of endoderm 8) Fox A1, 9) Cdx1; Key regulators of TEC development 10) Eya1, 11) Pax9, 12) FoxN1; Proteins typically expressed on TECs 13) EpCAM, 14) MHCII; Notch ligands expressed on TECs 15) Dll1, 16) Dll4, 17) Jag1, 18) Jag2; Wnts expressed by TECs 19) Wnt4, 20) Wnt10b; housekeeping control 21) HPRT. These results are representative of 5 independent experiments with 2 distinct TMSC lines performed from passage 4 to 7.
C. Comparison of Gene expression in sorted TEC subsets and TMSC7-10 at P16. Total RNA was isolated from TEC subsets sorted to >95% purity together with the clonal TMSC7-10 cell line. Quantitative PCR was then performed using a Taqman assay for the TEC specific markers Foxn1 and EpCAM as well as the stem cell markers Nanog, Oct4 and Sox2. All results were normalized to 18SrRNA and compared to the MHCII^int^ EpCAM^hi^ TEC subset using the ΔΔCt method.
(TIF)
