# Supplementary figures and images for: Label Retention Identifies a Multipotent Mesenchymal Stem Cell-Like Population in the Postnatal Thymus
Source: PLoS One. 2013 Dec 10;8(12):e83024. doi: 10.1371/journal.pone.0083024 (PMC3858364; doi:10.1371/journal.pone.0083024)

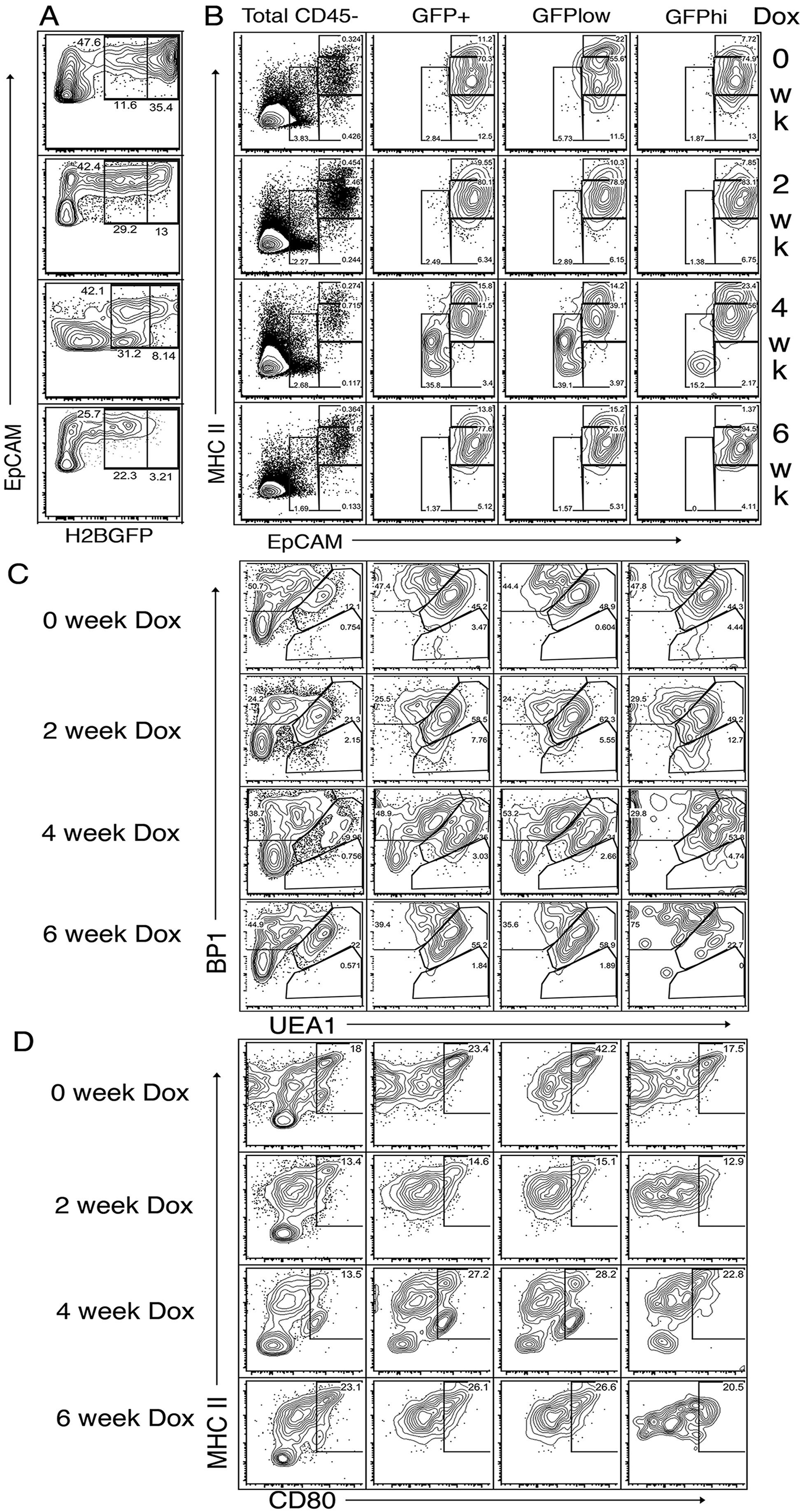

Supplement: Figure S1 — Cell Surface Profile of H2BGFP LRCs. A. Gating strategy for analysis of LRCs based on EpCAM and H2BGFP expression during 0-6 week Dox feeding time course. Gate frequencies from left to right show total H2BGFP+, H2BGFPlo and H2BGFPhi, respectively. B. Characterization of changes EpCAM and MHCII expression in total CD45- stroma, total GFP+, GFPlow and GFPhi subsets, every 2 weeks during a 6-week Dox time course. C. Characterization of changes in the frequency of MHCII versus CD80 in total CD45- stroma, total GFP+, GFPlow and GFPhi subsets, every 2 weeks during a 6-week Dox time course. D. Characterization of changes EpCAM and MHCII expression in total CD45- stroma, total GFP+, GFPlow and GFPhi subsets, every 2 weeks during a 6-week Dox time course. (TIF) [file pone.0083024.s001.tif]

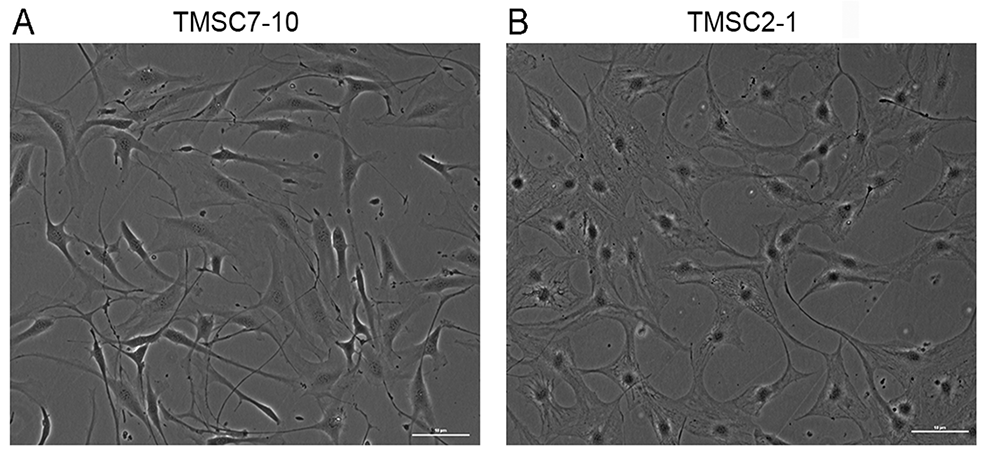

Supplement: Figure S2 — Morphology of TMSC lines in culture. A. Phase image of TMSC7-10 at P3; B. Phase image of TMSC2-1at P12. (TIF) [file pone.0083024.s002.tif]

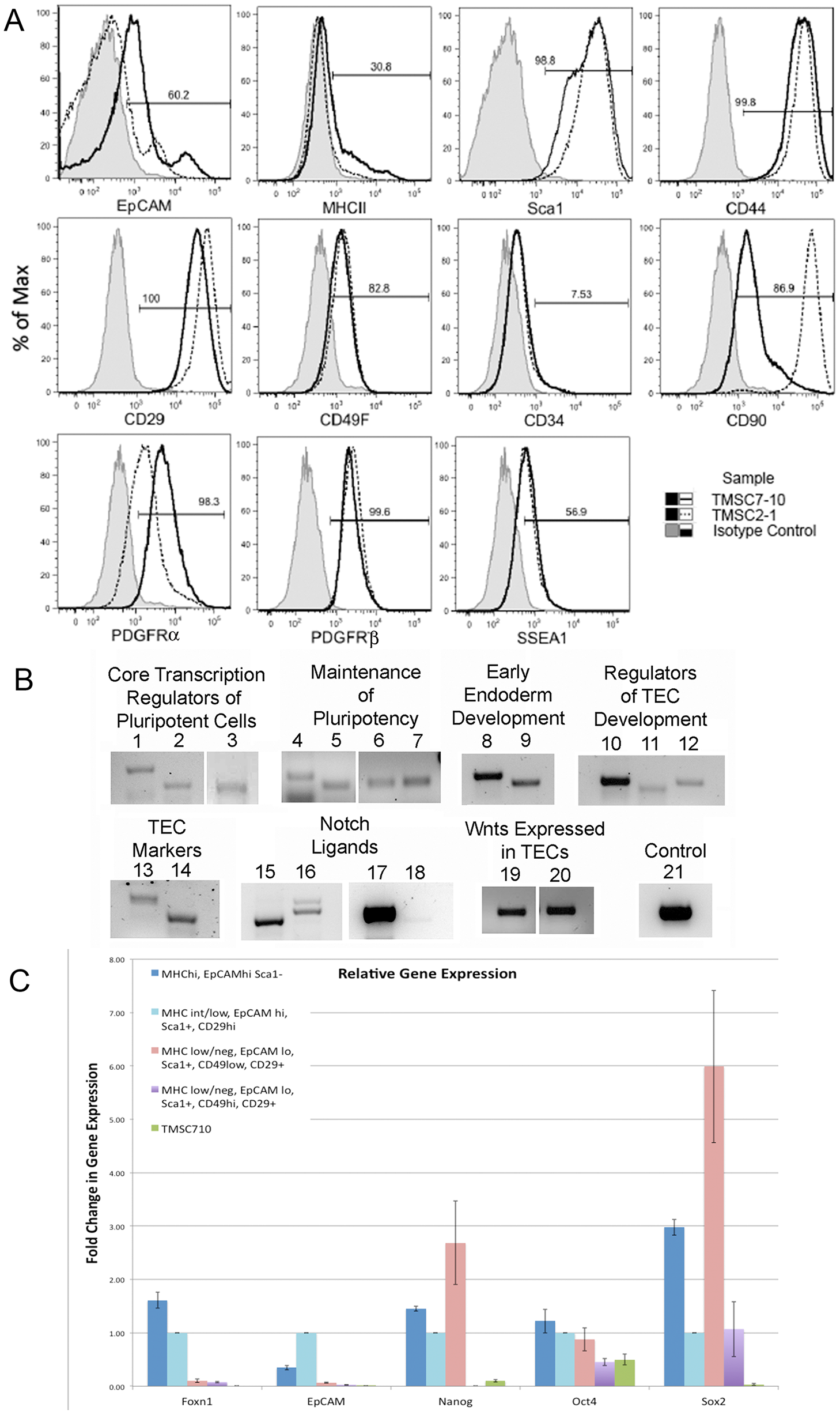

Supplement: Figure S3 — Gene Expression Profile of clonal TMSC lines. A. Clonal TMSC lines exhibit a surface profile similar to mesenchymal stem cells. Cell surface profile of TMSC7-10 and TMSC2-1 cell lines after 10 passages. For each antibody overlay, the grey filled histogram shows isotype control antibody staining and the solid and dotted black histograms shows staining with the specific antibody for the TMSC7-10 and TMSC2-1 cell lines, respectively. B.. Rt-PCR analysis of RNA isolated from TMSC7. Rt-PCR analysis of RNA isolated at P7 from TMSC7 revealed expression of core transcription regulators of pluripotent cells 1) Nanog, 2) Oct4 3) Sox2; Genes involved in the maintenance of pluripotency 4) Foxd3, 5) Lgr5, 6) Dppa3, 7) Utf1; transcription factors involved in early development of endoderm 8) Fox A1, 9) Cdx1; Key regulators of TEC development 10) Eya1, 11) Pax9, 12) FoxN1; Proteins typically expressed on TECs 13) EpCAM, 14) MHCII; Notch ligands expressed on TECs 15) Dll1, 16) Dll4, 17) Jag1, 18) Jag2; Wnts expressed by TECs 19) Wnt4, 20) Wnt10b; housekeeping control 21) HPRT. These results are representative of 5 independent experiments with 2 distinct TMSC lines performed from passage 4 to 7. C. Comparison of Gene expression in sorted TEC subsets and TMSC7-10 at P16. Total RNA was isolated from TEC subsets sorted to >95% purity together with the clonal TMSC7-10 cell line. Quantitative PCR was then performed using a Taqman assay for the TEC specific markers Foxn1 and EpCAM as well as the stem cell markers Nanog, Oct4 and Sox2. All results were normalized to 18SrRNA and compared to the MHCIIint EpCAMhi TEC subset using the ΔΔCt method. (TIF) [file pone.0083024.s003.tif]
